# Impact of seasonal changes on root-associated microbial communities among phreatophytes of three basins in desert ecosystem

**DOI:** 10.3389/fpls.2025.1554879

**Published:** 2025-07-04

**Authors:** Yulin Zhang, Yi Du, Zhaobin Mu, Waqar Islam, Fanjiang Zeng, Norela C. T. Gonzalez, Zhihao Zhang

**Affiliations:** ^1^ Xinjiang Key Laboratory of Desert Plant Roots Ecology and Vegetation Restoration, Xinjiang Institute of Ecology and Geography, Chinese Academy of Sciences, Urumqi, China; ^2^ State Key Laboratory of Ecological Safety and Sustainable Development in Arid Lands, Xinjiang Institute of Ecology and Geography, Chinese Academy of Sciences, Urumqi, China; ^3^ Cele National Station of Observation and Research for Desert-Grassland Ecosystems, Hotan, China; ^4^ College of Ecology and Environmental, Xinjiang University, Urumqi, China; ^5^ University of Chinese Academy of Sciences, Beijing, China; ^6^ State Key Laboratory of Advanced Environmental Technology and Guangdong Key Laboratory of Environmental Protection and Resources Utilization, Guangzhou Institute of Geochemistry, Chinese Academy of Sciences, Guangzhou, China; ^7^ College of Forestry, Central South University of Forestry and Technology, Changsha, Hunan, China

**Keywords:** seasonal dynamics, desert plants, soil nutrients, microbial composition, soil microbiome

## Abstract

Seasons often alter climate conditions and affect nutrient cycling by altering plant physiology and microbial dynamics. Plant growth and health depend on a symbiotic relationship with root microbes, however, the root-associated microbiota is key to plant evolution and ecosystem function. Seasonal changes in root-associated microbiome diversity and composition of desert plants are vital for understanding plant adaptation in desert ecosystems. We employed high-throughput sequencing to investigate the seasonal dynamics of root-associated microbial communities, including the root endosphere (RE), rhizosphere soil (RS), and bulk soil (BS), across three basins in Xinjiang, China: Turpan, Tarim, and Dzungaria. *Proteobacteria* dominated bacterial communities in different seasons, while *Ascomycota* prevailed in fungi. The spring and summer conditions favor greater microbial differentiation. The RE, RS, and BS bacterial communities in May (spring) showed a noticeable absence of highly connected nodes within and between modules. However, the opposite trend was observed in July (summer) and September (autumn). The community assembly of root-associated microbiome (bacteria and fungi) in different seasons primarily followed a random process. Random forest analysis found that seasonal variations in RE bacterial communities were primarily influenced by scattered radiation, while fungal communities were mainly affected by soil available potassium. Environmental factors affect the BS bacterial community more than the fungal community across different seasons. A structural equation model revealed temperature and precipitation’s direct effects on microbial communities, mediated by soil and root nutrient availability. Soil pH and EC predominantly affected root bacterial communities, not fungal communities. The fungal community within the RE was found to be directly influenced by seasonal shifts, whereas the RS fungal community composition was significantly impacted by changes in precipitation patterns driven by seasonal variation. The climate seems to be a crucial factor in influencing the dynamic of the root microbiome in desert plants, surpassing the influence of soil and root nutrient availability. This study underscores seasonal root-associated microbiome variations and their important roles in desert ecosystem functions.

## 1 Introduction

Plant roots harbor a distinctive microbiome comprising soil-derived microorganisms (Chen et al., 2019; Zhang et al., 2018). These microorganisms form a symbiotic relationship with the plant, which is significant for the plant because it affects its growth and nutrient absorption throughout its life cycle (Domeignoz-Horta et al., 2020; Oldroyd and Leyser, 2020). Although seasonal climate changes can influence plant communities (Fraterrigo and Rusak, 2008), there is a lack of studies investigating seasonal variations in root-associated microbiomes (Hannula et al., 2019). Microbial seasonality has not been fully understood due to neglect of various microbial groups (Regan et al., 2014; Wang et al., 2018). Furthermore, few studies concurrently investigate microbial variations across different root compartments (Monson et al., 2006; Wu et al., 2016). Recent studies have illuminated root-associated microbiomes as sensitive bioindicators to environmental changes, exhibiting seasonal community shifts across diverse ecosystems (Manzoni et al., 2012). These changes may affect soil biogeochemical cycles (Siles et al., 2016). The impact of elevated temperatures on soil microbial physiology and community composition has been extensively explored through short-term warming experiments over the past few decades (Jansson and Hofmockel, 2020). However, soil microbial communities do not simply respond passively to temperature fluctuations; they also exhibit adaptive responses to prolonged warming (Bradford et al., 2008). Consequently, delving into the seasonal dynamics of root-associated microbiomes promises a comprehensive comprehension of how climate change impacts biogeochemical processes in desert ecosystems.

Despite numerous research studies into the seasonal fluctuations of root-associated microbiome, a coherent understanding remains elusive, lacking consensus on influencing factors (Tan et al., 2014; Hannula et al., 2019). Seasonality is common in natural ecosystems, indicating the concurrent fluctuation of different environmental elements like temperature, rainfall, and soil characteristics (Zhang et al., 2023; Howe et al., 2023). Studies have demonstrated that late-season precipitation significantly increases the biomass of arbuscular mycorrhizal fungi (AMF) and markedly alters the relative abundance of dominant genera within AMF and protist community (Yu et al., 2024). In addition, the relative abundance of major bacterial phyla such as Actinobacteria, Firmicutes, and Proteobacteria has been found to correlate strongly with annual mean temperature and precipitation at the watershed scale. However, seasonal variation—rather than spatial heterogeneity—emerged as the primary driver of the composition of soil antibiotic resistance genes (Xiang et al., 2021). Despite the widely recognized impact of seasonality on ecological processes, our understanding of how it fully affects the assembly of root microbial communities remains inadequate (White and Hastings, 2020). Previous studies have shown notable variations throughout the seasons in microbial populations and their functional categories (He et al., 2021), especially in agricultural and water environments (Grady et al., 2019; He et al., 2020; Maurice et al., 2024a). A comparison between ecological floating beds planted with *Cyperus involucratus* Rottb., *Thalia dealbata* Fraser, and *Iris tectorum* Maxim, and a control group (static water) revealed that root-associated microbial communities exhibited their highest abundance and diversity in autumn. These communities were dominated by taxa such as *Proteobacteria, Actinobacteriota, Cyanobacteria, Chloroflexi, Firmicutes, Bacteroidota*, and *Acidobacteriota*, highlighting a distinct seasonal pattern of microbial proliferation (Felipe et al., 2021; Zhang et al., 2022a). Distinct soil fungal community composition differences were found among forest types and seasons (Xie and Yin, 2022). Nevertheless, the impact of seasonal fluctuations on the structure of root-associated microbial communities in arid ecosystems has not been widely explored (Eilers et al., 2010). These microbial communities are affected by a mixture of climatic, soil physicochemical, and biological factors, among which temperature and precipitation are of vital importance (Ibekwe et al., 2020). Seasonal shifts influence soil physicochemical attributes and resource quality, resulting in variations in microbial composition (Sharhabil et al., 2023; Fan et al., 2023). Several studies have investigated the relationship between environmental factors and soil bacterial diversity across 90 different habitats in China’s temperate desert region. These studies indicate that soil bacterial α and beta diversity are primarily driven by abiotic and spatial factors, followed by plant-related influences (Wang et al., 2020). Among these abiotic factors, soil pH has emerged as a key determinant of bacterial diversity and community composition. It has a stronger influence than spatial and climatic (biome-related) variables in shaping microbial patterns (Zhou et al., 2024). In a separate study conducted in Michigan, USA, soils were sampled monthly from May to November across replicated plots of three land-use types: conventional row-crop agriculture, reduced-input row-crop agriculture, and early successional grasslands. Results showed that α-diversity exhibited stronger temporal variability driven by sampling month than by land-use type. In contrast, β-diversity patterns remained relatively stable across land-use types throughout the 7-month period, suggesting that the timing of sampling is less critical when assessing community composition across different land uses (Rasche et al., 2011; Lauber et al., 2013). Furthermore, the structure of soil microbial communities can also vary in response to seasonal fluctuations in plant activity and carbon inputs (Islam et al., 2023; Gao et al., 2024). Mostly, Plants selectively cultivate specific microbial communities in the rhizosphere through root exudates, influencing the surrounding soil’s physical and chemical characteristics (Wang et al., 2022). Microbial responses to seasonal changes differ between the rhizosphere and bulk soil, with significant differences among different microbial categories (Xu et al., 2023; Markus et al., 2024).

Desert ecosystems, found in arid and semi-arid regions, are defined by extreme water scarcity, high temperatures, and limited nutrients (Ibáñez et al., 2007; Tariq et al., 2022; Islam et al., 2024a). Desert vegetation has evolved special adaptation mechanisms to cope with these extreme circumstances (Zhang et al., 2022b; Islam et al., 2024b; Gao et al., 2024). *Calligonum caput-medusae* Schrenk., *Alhagi* sp*arsifolia* Shap., and *Tamarix ramosissima* Ledeb., are key desert plants that contribute to ecological stability by preventing wind erosion and stabilizing sand, while also serving as valuable forage to support regional animal husbandry (Zhang et al., 2020; Islam et al., 2024c). The study collected root endosphere (RE), rhizosphere soil (RS), and bulk soil (BS) samples from three key desert plants—*C. caput-medusae*, *A.* sp*arsifolia*, and *T. ramosissima*—in Xinjiang, China. These species are crucial for the stability and sustainability of the desert ecosystem. We investigated and analyzed how seasonal changes affect the bacterial and fungal communities associated with the roots using high-throughput amplicon sequencing on the Illumina HiSeq platform. This study investigated the composition and diversity of root-associated microbiomes to uncover their distinct seasonal dynamics and the underlying influences of soil and plant nutrients. We hypothesized that: (i) the community composition, diversity, co-occurrence patterns, and assembly processes of root-associated microbiomes—including root endosphere (RE), rhizosphere (RS), and bulk soil (BS) compartments—would vary across seasons; and (ii) specific environmental factors would significantly influence the composition and diversity of these microbiomes in different seasons. Our findings shed light on the seasonal shifts in microbial communities associated with key desert plants and the environmental drivers shaping these patterns. This research provides critical insights into microbially mediated ecological adaptations, supporting strategies for enhancing the resilience and sustainable management of desert ecosystems under arid and stressful conditions.

## 2 Materials and methods

### 2.1 Study area overview, experimental design, and sample collection

The experiment was carried out at three long-term desert locations {Xinjiang Institute of Ecology and Geography, Chinese Academy of Sciences (XIEG, CAS)}: Cele and Mosuowan desert research station (CL and MSW) and Turpan desert botanical garden (TLF). The climate data are from the Cele, Turpan, and Mosuowan desert research stations. For detailed information, see **Tables 1**, **2**; **Supplementary Figure S1**.

**Table 1:** Geographic and climatic characteristics in the three study sites.

Characteristics		Site		
		Cele (CL)	Mosuowan (MSW)	Turpan (TLF)
Geographic	Latitude (°N)	35°00′57″	45°07′27″	42°51′59″
	Longitude (°E)	80°43′45″	86°01′31″	89°12′01″
	Altitude (m)	1318	346	-105 m ~ -76 m
Climatic	MAT (°C)	11.9	6.6	13.9
	MAP (mm)	35.1	117.0	16.4
	PEP (mm)	2595.3	1979.5	3000
	AI	0.01	0.06	0.005
Soil type	ST	aeolian sandy soil	grey desert soil	grayish brown desert soil

MAT, mean annual temperature; MAP, mean annual precipitation; PEP, potential evapotranspiration; AI, aridity index, calculated as AI = MAP/PEP. ST, soil type.

**Table 2:** Climate factors in three years (mean).

Site	Season	Temp (°C)	Prep (mm)	Atm (hPa)	WS (m/s)	WD (°)	DR (w/m^2^)	SR (w/m^2^)	HR (w/m^2^)	Hum (%)
Cele	May	23.64	0.0015	854.99	4.13	213.63	288.37	94.73	306.46	26. 20
	July	29.90	0.0055	851.74	3.46	195.02	293.75	92.55	307.92	23.66
	September	23.55	0.00012	857.97	3.06	208.06	308.12	63.34	253.50	24.58
Mosuowan	May	27.08	0.015	969.79	4.21	186.71	261.38	99.84	281.16	27.64
	July	30.31	0.0060	964.89	4.04	199.54	249.55	107.84	284.32	27.36
	September	23.19	0.018	973.43	3.38	169.83	239.25	64.30	197.62	31.99
Turpan	May	29.89	0.00096	1011.62	2.37	196.76	252.62	103.63	281.20	20.76
	July	34.00	0.0011	1005.56	2.49	203.89	238.97	110.22	281.27	23.27
	September	27.98	0.00056	1014.75	1.68	197.45	295.64	63.48	228.22	21.45

Temp, temperature; Prep, precipitation; Atm, atmospheric pressure; WS, wind speed; WD, wind direction; DR, direct radiation; SR, scattered radiation; HR, horizontal radiation; Hum, air relative humidity.

Four homogeneous quadrats, each measuring approximately 30 m × 30 m, were selected at each of the three study fields (stations). Each quadrat included three representative desert plant species—*C. caput-medusae*, *A.* sp*arsifolia*, and *T. ramosissima*—that thrive naturally. In total, twelve research blocks were chosen. Fieldwork was conducted at three long-term observation stations in May (spring), July (summer), and September (autumn) of 2022. The samples were divided into RE, RS, and BS compartments (Edwards et al., 2015). Root and soil samples representing RE, RS, and BS were collected from a range of desert plant species at depths of 0 to 2 meters. Both soil and root samples were collected at corresponding depths to ensure consistent sampling across compartments. The BS, which was loosely adhered to fine roots, was used for evaluating its physical and chemical properties. We used a sterile brush to gather RS, closely associated with fine roots (≤ 2 mm), by collecting soil from the roots. The collected soil was then placed in individual sterile centrifuge tubes. The root samples were immersed in 75% alcohol, oscillating them for three rounds, each lasting 15 secondsRoots were thoroughly cleaned by washing with sterile water on a vortex oscillator for three one-minute intervals to remove adhering soil particles. The cleaned roots were then cut into small fragments and placed into sterile containers. In total, 324 samples were collected throughout 2022, encompassing three plant species, three compartments (RE, RS, and BS), multiple basins, and three seasons, with four biological replicates per group. All samples were immediately stored at –80 °C in an ultra-low temperature freezer to preserve microbial integrity prior to DNA extraction.

### 2.2 DNA extraction, polymerase chain reaction, and Illumina sequencing

To extract total soil genomic DNA from 0.5 g of BS and RS, the DNA extraction kit manufactured by Qiagen, Inc. in the Netherlands was utilized. RE samples were prepared by pulverizing 0.4 g with liquid nitrogen, followed by extracting total microbial DNA using the DNeasy Plant Maxi Kit (Qiagen, Netherlands). PCR amplification targeted the bacterial 16S rRNA gene V3-V4 region {341F (5’-CCTAYGGGRBGCASCAG-3’) and 806R (5’-GGACTACNNGGGTATCTAAT-3’)}and the fungal ITS (Internal Transcribed Spacer) 1–5 F region {5-1737F (5’-GGAAGTAAAAGTCGTAACAAGG-3’) and 2-2043R (5’-GCTGCGTTCTTCATCGATGC-3’)}. Each sample was distinguished from the dismounting data using its Barcode and PCR primer sequences. After removing barcode and primer sequences, the reads for each sample were merged using FLASH (v1.2.11) (Magoč and Steven, 2011), generating raw tag sequences. These raw tags were then processed using FASTP (v0.23.1) with stringent quality filtering parameters to produce high-quality clean tags (Bokulich et al., 2013). o further improve sequence quality, chimeric sequences were identified and removed using VSEARCH (v2.16.0) by aligning the tags to a reference species annotation database. The resulting high-quality, non-chimeric sequences were designated as effective tags for downstream analysis (Rognes et al., 2016).

Additional sequence filtering was performed using the QIIME-II software (v202202) (Caporaso et al., 2010). DADA2 plugin was used to control the quality, denoise, splicing, and removal of chimerism to generate amplicon sequence variants (ASVs) (Callahan et al., 2016). Using the RDP (Ribosomal Database Project) classifier at a 70% confidence threshold, the Mothur method, and SSUrRNA database from Silva version 138.1 with a 0.8–1 threshold range, bacterial ASVs were identified (Wang et al., 2007; Quast et al., 2013). Fungal ASVs were classified using the UNITE database (Altschul et al., 1990). The original sequencing data was stored at the National Center for Biotechnology Information (NCBI) under the number PRJNA1024038. The dilution curve is shown in **Supplementary Figure S2**.

### 2.3 Soil’s physical and chemical properties

The oxidation method using K_2_Cr_2_O_7_-H_2_SO_4_ was employed to analyze the contents of root and soil organic carbon (ROC and SOC), whereas the total nitrogen (TN) contents were quantified using the Kjeldahl Nitrogen Analyzer (K1160, produced by Jinan Hanon Instruments Co. Ltd., China) (Hooper and Vitousek, 1998; Bao, 2000). Available nitrogen (AN) was assessed through the alkali hydrolysis method, while total phosphorus (TP) and total potassium (TK) were analyzed with the Inductively Coupled Plasma-Optical Emission Spectrometer (iCAP 6300, Thermo Elemental, USA) following sample dissolution in concentrated nitric acid (Neff et al., 2005; Warra et al., 2015; Lu et al., 2015). Available phosphorus (AP) was extracted by combining hydrochloric acid and ammonium fluoride, followed by analysis using colorimetric method with ascorbic acid molybdate on a continuous-flow auto-analyzer manufactured by Bran and Luebbe in Germany (Olsen and Sommer, 1982). The NH_4_OAc extraction method was applied to ascertain the available potassium (AK) content (Warra et al., 2015). Soil pH levels were measured using a pH meter (PHSJ-6 L, INESA Scientific Instrument Co. Ltd., China) with a 1:2.5 soil-to-water ratio, and electrical conductivity (EC) was assessed with an EC meter (DDSJ-319 L, INESA Scientific Instrument Co. Ltd., China) at a 1:5 soil to water ratio (Bao, 2000).

### 2.4 Metrological/climate data

Meteorological data were collected from the National Meteorological Science Data Center (https://data.cma.cn/) for three sites—Cele, Turpan, and Mosuowan—managed by the Chinese Academy of Sciences. The dataset included temperature (Temp), precipitation (Prep), relative humidity (Hum), atmospheric pressure (Atm), wind speed (WS), wind direction (WD), direct radiation (DR), horizontal radiation (HR), and scattered radiation (SR) (**Table 2**).

### 2.5 Statistical analyses

Statistical analysis was performed using R version 4.1.0 (R Core Team, 2021). The Shapiro-Wilk normality test was used to check the normality of the raw data. The findings were primarily presented using packages like ‘ggplot2’, ‘microeco’, ‘vegan’, ‘corrplot’, ‘randomForest’, ‘rfPermute’, and ‘iCAMP’ (Hahsler et al., 2008; Wei and Simko, 2013; Archer, 2016; Oksanen et al., 2017). A one-way ANOVA was conducted to assess the seasonal impact on the physical and chemical properties of roots and soil. The α-diversity (Chao1, Shannon-Wiener, Pielou_e, and Simpson index) at the ASVs level was calculated using QIIME2. For the calculation of β diversity, the vectorized ASVs matrix based on the Bray-Curtis distance was adopted. The impact of season on the top 10 relative abundances, Observed_OTUs number, and both α-diversity and β-diversity was assessed using one-way and two-way ANOVA. The agricolae package was employed to analyze sources of variation through the least significant difference (LSD) test (Mendiburu, 2020).

To assess community assembly processes, we employed a phylogenetic bin-based null model analysis framework using the iCAMP package (Stegen et al., 2015; Ning et al., 2020). This framework quantifies the relative contributions of deterministic and stochastic processes, with higher absolute values of the standardized effect size (SES) indicating a stronger influence of deterministic forces. First, phylogenetic signals were assessed using a phylogenetic Mantel correlogram to determine whether phylogenetic turnover could reliably inform ecological inference within the system (Dini-Andreote et al., 2015). Second, the beta mean nearest taxon distance (βMNTD) was recalculated to generate a null distribution, obtained through 999 random permutations. The deviation of the observed βMNTD from the mean of this null distribution, expressed in standard deviation units, was calculated as the beta nearest taxon index (βNTI). The βNTI value less than –2 or greater than +2 indicates significantly lower or higher phylogenetic turnover than expected by chance, respectively, suggesting the dominance of deterministic processes (Stegen et al., 2012).

Co-occurrence networks were constructed using Gephi software based on Spearman’s correlation matrices, retaining only correlations with an absolute coefficient above 0.7 and a false discovery rate (FDR)-adjusted p-value below 0.001. The topological features of microbial network modules, including within-module connectivity (Zi) and among-module connectivity (Pi), were used to classify nodes into four categories: module hubs (high connectivity within modules; Zi > 2.5, Pi < 0.62), connectors (high connectivity between modules; Zi < 2.5, Pi > 0.62), network hubs (high connectivity both within and across modules; Zi > 2.5, Pi > 0.62), and peripherals (low connectivity; Zi < 2.5, Pi < 0.62) (Deng et al., 2012).

To identify key environmental drivers influencing root microbial community composition, random forest analysis was performed using the first principal coordinate axis derived from principal coordinate analysis (PCoA) of amplicon sequence variant (ASV)-level data as the response variable. The ‘randomForest’ and ‘rfPermute’ R packages were employed to assess variable importance, measured by the percentage increase in mean squared error (%IncMSE), with significance levels calculated accordingly.

Structural equation modeling (SEM) was applied to explore the pathways through which climate, soil, and plant factors affect microbial communities in desert plant roots. PCoA was used to reduce dimensionality of soil available nutrients (nitrogen, phosphorus, potassium), root nutrients (organic carbon, total nitrogen, total phosphorus, total potassium), and microbial community composition across root compartments (root endosphere, rhizosphere soil, and bulk soil), with the first axis scores serving as variables in SEM analyses conducted in Amos-24 software. Model fit was evaluated based on multiple criteria: chi-square (χ²) with *P* > 0.05 indicating good fit; chi-square/degrees of freedom (χ²/df) between 1 and 3; root mean square error of approximation (RMSEA) below 0.05; comparative fit index (CFI) and goodness of fit index (GFI) both exceeding 0.90; and Akaike information criterion (AIC), where lower values indicate better fit and facilitate model comparison (Lange et al., 2015; Fan et al., 2016).

## 3 Results

### 3.1 Effects of seasonal changes on soil and root physical and chemical properties

In May, the soil TK content was significantly lower than that in July and September. The root ROC in May was also significantly lower than that in July. Throughout the growth seasons of May, July, and September, there was no significant difference between the soil properties (pH, EC, SOC, TN, TP, AN, AP, and AK) and root nutrient contents (TN, TP, and TK) (**Table 3**).

**Table 3:** Seasonal changes of soil physicochemical properties and root nutrient content of desert plants.

Factor	Index	Spring	Summer	Autumn
Soil	SOC (g•kg^-1^)	2.81 ± 0.30a	2.8 ± 0.26a	2.62 ± 0.30a
	TN (g•kg^-1^)	0.23 ± 0.03a	0.23 ± 0.03a	0.22 ± 0.03a
	TP (g•kg^-1^)	0.75 ± 0.04a	0.75 ± 0.03a	0.75 ± 0.04a
	TK (g•kg^-1^)	18.97 ± 0.29b	20.59 ± 0.34a	20.21 ± 0.36a
	AN (mg•kg^-1^)	14.37 ± 1.62a	16.56 ± 1.61a	14.9 ± 1.51a
	AP (mg•kg^-1^)	3.25 ± 0.36a	3.57 ± 0.37a	3.39 ± 0.31a
	AK (mg•kg^-1^)	265.67 ± 20.8a	294.83 ± 29.93a	286.42 ± 21.92a
	pH	8.72 ± 0.04a	8.74 ± 0.06a	8.69 ± 0.05a
	EC (uS•cm^-1^)	969.55 ± 188.87a	1252.46 ± 235.13a	1089.86 ± 181.74a
Root	ROC (g•kg^-1^)	453.53 ± 3.82b	465.26 ± 3.66a	462.07 ± 1.92ab
	TN (g•kg^-1^)	9.05 ± 0.61a	7.65 ± 0.50a	7.15 ± 0.71a
	TP (g•kg^-1^)	0.5 ± 0.08a	0.44 ± 0.04a	0.41 ± 0.05a
	TK (g•kg^-1^)	4.54 ± 0.44a	3.88 ± 0.27a	4.33 ± 0.37a

Different lowercase letters (a, b, and c) indicate that the different seasons have significant differences (LSD test, *P* < 0.05). SOC, soil organic carbon (g•kg^-1^); ROC, root organic carbon (g•kg^-1^); TN, total nitrogen (g•kg^-1^); TP, total phosphorus (g•kg^-1^); TK, total potassium (g•kg^-1^); AN, available nitrogen (mg•kg^-1^); AP, available phosphorus (mg•kg^-1^); AK, available potassium (mg•kg^-1^); EC, electrical conductivity (uS•cm^-1^). Spring (May), Summer (July), and Autumn (September).

### 3.2 Seasonal variations influence the abundance and Observed_OTUs number in root microbial communities

**Figure 1:**
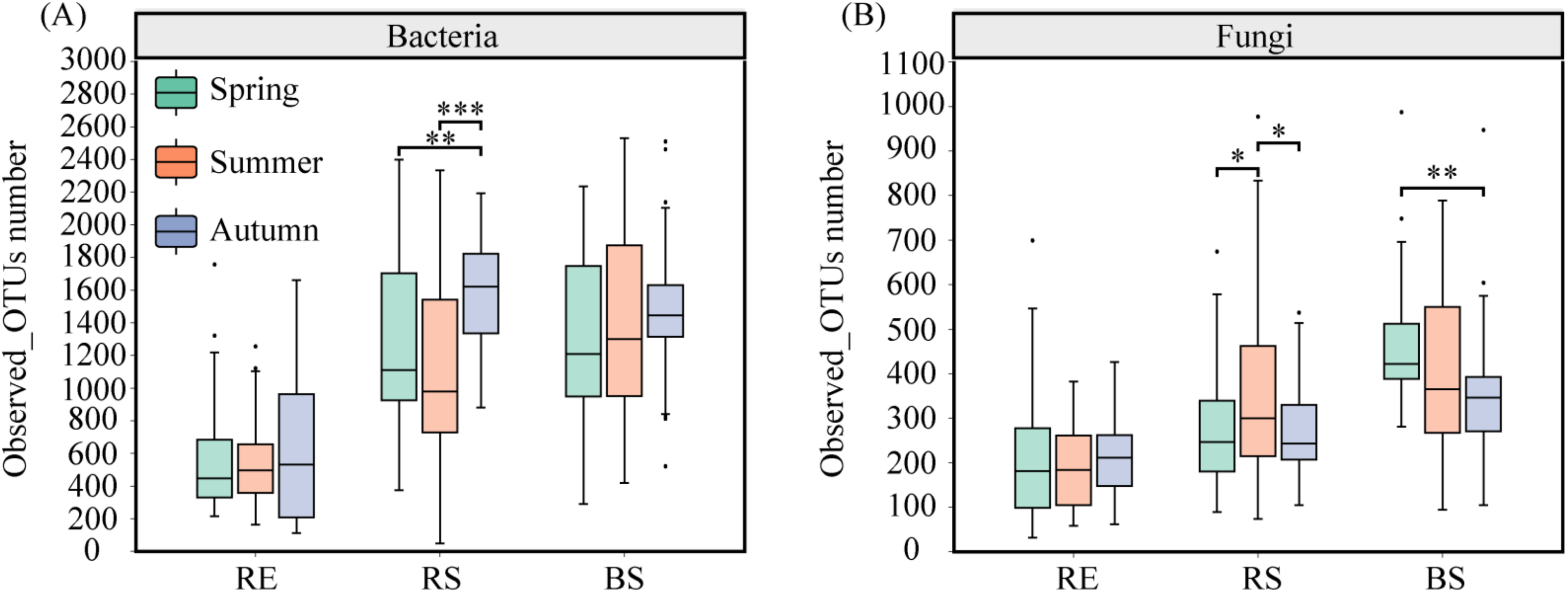
The Observed_OTUs number of root-associated dominant bacterial and fungal of desert plants. **(A)** the Observed_OTUs number of root-associated dominant bacterial of desert plants. **(B)** the Observed_OTUs number of root-associated dominant fungal of desert plants. RE, root endosphere; RS, rhizosphere soil; BS, bulk soil. Spring (May); Summer (July); and Autumn (September). Significance codes, *P < 0.05; **P < 0.01; ***P< 0.001.

Seasons significantly affected the root bacterial Observed_OTUs number (*p* < 0.01). Root partitioning significantly affected the root bacterial and fungal Observed_OTUs number (*p* < 0.001). Season and root partitioning interaction significantly influenced fungal Observed_OTUs number (*p* < 0.01) (**Table 4**). The RS bacterial Observed_OTUs number in September was significantly greater than in May and July (*p* < 0.05) (**Figure 1A**). The RS fungal Observed_OTUs number was significantly higher in July compared to May and September, while the BS fungal Observed_OTUs number were significantly more numerous in May than in September (*p* < 0.05) (**Figure 1B**).

**Table 4:** Variance analysis of α diversity in typical desert plants by different seasons and root partitioning.

Factor	Index	Season	Root partitioning	Season×Root partitioning
Bacteria	Observed_OTUs number Chao1	5.90** 5.72**	97.42*** 95.81***	1.75 1.72
	Shannon-Wiener	3.86*	152.74***	2.80*
	Pielou_e	3.26*	148.76***	2.83*
	Simpson	3.26*	84.37***	2.45*
Fungi	Observed_OTUs number Chao1	2.45 3.18*	57.59*** 57.52***	3.90** 3.81**
	Shannon-Wiener	2.61	147.33***	3.66**
	Pielou_e	4.99**	137.81***	2.68*
	Simpson	6.98***	108.81***	2.20

*P < 0.05; **P < 0.01; ***P < 0.001.

**Figure 2:**
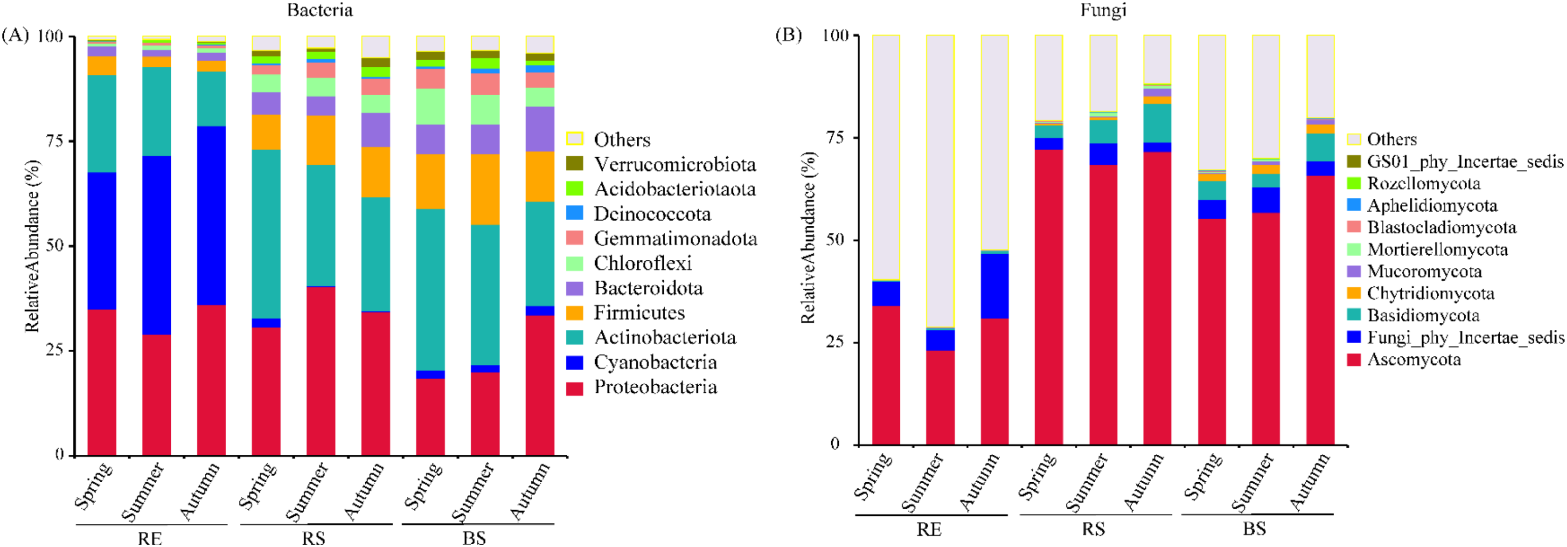
The abundances of root-associated dominant bacterial and fungal of desert plants. **(A)** the relative abundance of dominant bacteria (top 10 phyla) from different seasons. **(B)** the relative abundance of dominant fungi (top 10 phyla) from different seasons. RE, root endosphere; RS, rhizosphere soil; BS, bulk soil. Spring (May); Summer (July); and Autumn (September).

For bacteria, seasons significantly affected the relative abundance of *Proteobacteria*, *Actinobacteriota*, *Bacteroidota*, *Chloroflexi*, and *Deinococcota* (*p* < 0.05). Root partitioning significantly affected the relative abundance of *Proteobacteria*, *Cyanobacteria*, *Verrucomicrobiota*, *Acidobacteriotaota*, *Deinococcota*, *Gemmatimonadota*, *Chloroflexi*, *Bacteroidota*, *Firmicutes*, and *Actinobacteriota* (*p* < 0.001). Season and root partitioning interaction significantly influenced the relative abundance of *Proteobacteria*, *Chloroflexi*, *Gemmatimonadota*, *Deinococcota*, *Acidobacteriotaota*, and *Verrucomicrobiota* (*p* < 0.05) (**Supplementary Table S1**). In RE, bacterial *Proteobacteria* show reduced relative abundance in July compared to May and September. In RS, the relative abundance of bacterial *Proteobacteria* exhibits a contrasting pattern. The relative abundance of BS bacterial *Proteobacteria* is greater in September compared to May and July (**Figure 2A**). The relative abundance of RE bacterial *Actinobacteriota* is lower in September compared to May and July (**Figure 2A**; **Supplementary Table S1**).

For fungi, seasons significantly affected the relative abundance of *Basidiomycota* and *Mortierellomycota* (*p* < 0.05). Root partitioning significantly affected the relative abundance of *Ascomycota*, *Fungi_phy_Incertae_sedis*, *Basidiomycota*, *Chytridiomycota*, *Mortierellomycota*, *Aphelidiomycota*, and *Rozellomycota* (*p* < 0.01). Season and root partitioning interaction significantly influenced the relative abundance of *Fungi_phy_Incertae_sedis*, *Basidiomycota*, and *Mortierellomycota* (*p* < 0.05) (**Supplementary Table S1**). For *Ascomycota* in RE and RS fungi, its relative abundance in July was lower than that in May and September. In BS fungi, *Ascomycota* showed a higher relative abundance in September compared to May and July (**Figure 2B**). For *Basidiomycota* in root compartments (RE, RS, and BS) compartments fungi, its relative abundance in September was higher than that in May and July (**Figure 2B**; **Supplementary Table S1**).

### 3.3 Seasonal variations influence the community assembly processes of root microbial communities

**Figure 3:**
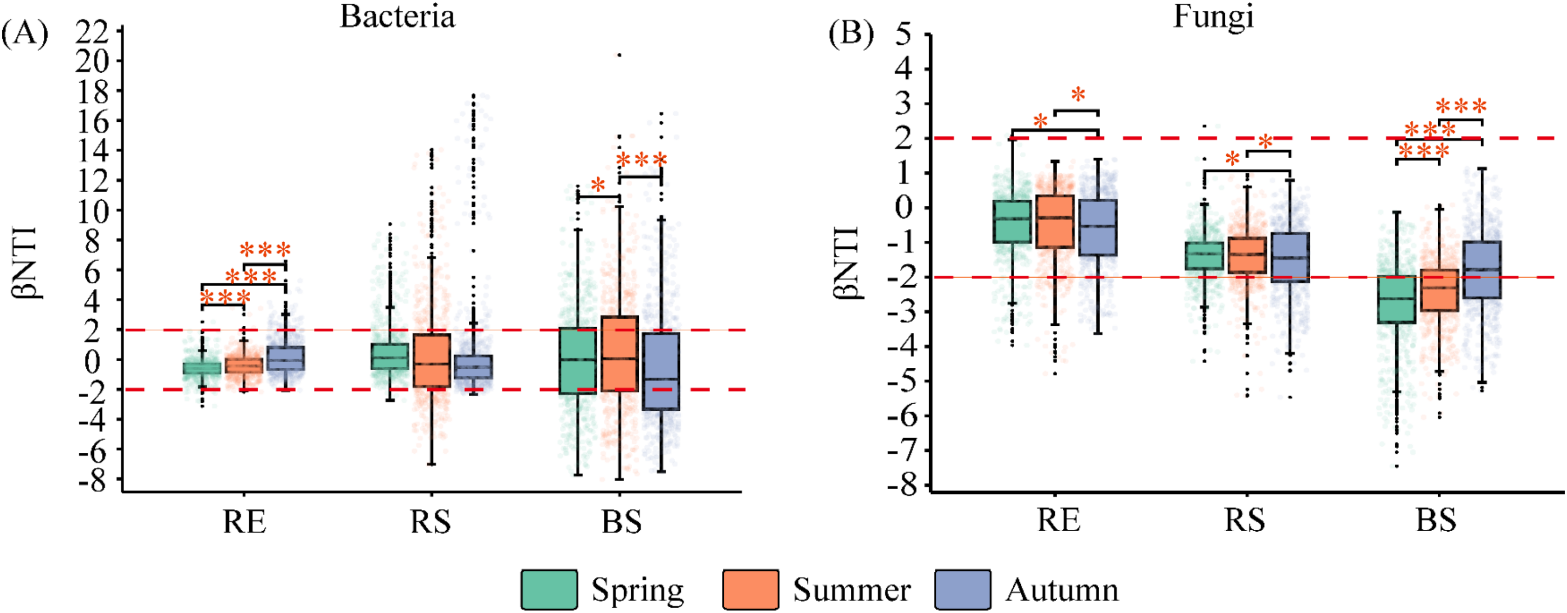
The β nearest taxon index (βNTI) of bacterial and fungal communities among season in desert plants. **(A)** the βNTI of bacterial communities among season in desert plants. **(B)** the βNTI of fungal communities among season in desert plants. RE, root endosphere; RS, rhizosphere soil; BS, bulk soil. Spring (May); Summer (July); and Autumn (September). Significance codes, *P < 0.05; ***P < 0.001.

The mantel correlogram revealed that both the RE, RS, and BS bacterial and fungal communities had significant phylogenetic signals (**Supplementary Figure S3**). The community assembly of root bacteria and fungi in different seasons primarily followed a random process. The BS bacterial community assembly in different seasons was primarily governed by a deterministic process. For fungi (BS), the community construction in May and July was dominated by a deterministic process, while in September, it was dominated by a random process (**Figures 3A, B**; **Table 5**).

**Table 5:** Community construction process of desert plant root microbial communities in different seasons.

Factor	Assembly process	Properties	RE				RS				BS		
			Spring	Summer	Autumn	Spring		Summer	Autumn	Spring		Summer	Autumn
Bacteria	Deterministic	HES (%)	0.16	0.63	7.14	12.70		22.38	9.37	26.19		31.90	22.70
		HOS (%)	2.06	0.63	0.16	0.63		22.70	4.44	29.05		26.83	42.22
		Sum (%)	2.22	1.27	7.30	13.33		45.08	13.81	55.24		58.73	64.92
	Stochastic	DL (%)	65.56	49.84	40.32	73.49		35.24	72.70	41.11		39.37	33.49
		HD (%)	0.79	1.75	0.63	2.86		1.27	2.54	0.63		0.48	0.32
		Drift (and other) (%)	31.43	47.14	51.75	10.32		18.41	10.95	3.02		1.43	1.27
		Sum (%)	97.78	98.73	92.70	86.67		54.92	86.19	44.76		41.27	35.08
Fungi	Deterministic	HES (%)	0.16	0.00	0.00	0.16		0.00	0.00	0.00		0.00	0.00
		HOS (%)	8.57	10.63	12.54	17.14		20.32	28.73	74.76		65.24	42.54
		Sum (%)	8.73	10.63	12.54	17.30		20.32	28.73	74.76		65.24	42.54
	Stochastic	DL (%)	36.19	51.75	58.10	71.43		62.70	55.87	24.76		26.67	55.24
		HD (%)	3.33	4.13	2.54	0.32		0.16	0.16	0.16		0.16	0.32
		Drift (and other) (%)	51.75	33.49	26.83	10.95		16.83	15.24	0.32		7.94	1.90
		Sum (%)	91.27	89.37	87.46	82.70		79.68	71.27	25.24		34.76	57.46

RE, root endosphere; RS, rhizosphere soil; BS, bulk soil. HES, Heterogeneous Selection; HOS, Homogeneous Selection; DL, Dispersal Limitation; HD, Homogenizing Dispersal. Spring (May), Summer (July), and Autumn (September).

For RE bacteria, deterministic processes in spring and summer were primarily driven by homogeneous selection (HOS), while stochastic processes were mainly dominated by dispersal limitation (DL). For RS bacteria, deterministic processes in spring and autumn were mainly governed by heterogeneous selection (HES), whereas random processes across spring, summer, and autumn were predominantly influenced by DL. In BS bacteria, deterministic processes in spring and autumn were largely attributed to HOS, with DL being the main random process throughout spring, summer, and autumn. For fungi in the RE, RS, and BS compartments, deterministic processes across all three seasons (spring, summer, and autumn) were primarily associated with HOS, while random processes were mainly driven by DL (**Table 5**).

The deterministic processes of bacteria (RE and RS) and fungi (RE) contributed more in July compared to May and September. For bacteria (BS), the deterministic process contributed less in July compared to May and September. For fungi (RS and BS), the deterministic process’s relative contribution in different seasons was highest in September, followed by July, and lowest in May (**Supplementary Figure S4**).

### 3.4 Seasonal variations influence the root microbial α and β diversity

Seasons significantly affected the root bacterial and fungal Chao1, Pielou_e, and Simpson indices (*p* < 0.05). Root partitioning significantly affected the root bacterial and fungal Chao1, Shannon-Wiener, Pielou_e, and Simpson indices (*p* < 0.05). Season and root partitioning interaction significantly influenced bacterial (Shannon-Wiener, Pielou_e, and Simpson) and fungal diversity (Chao1, Shannon-Wiener, and Pielou_e) (*p* < 0.05) (**Table 4**).

**Figure 4:**
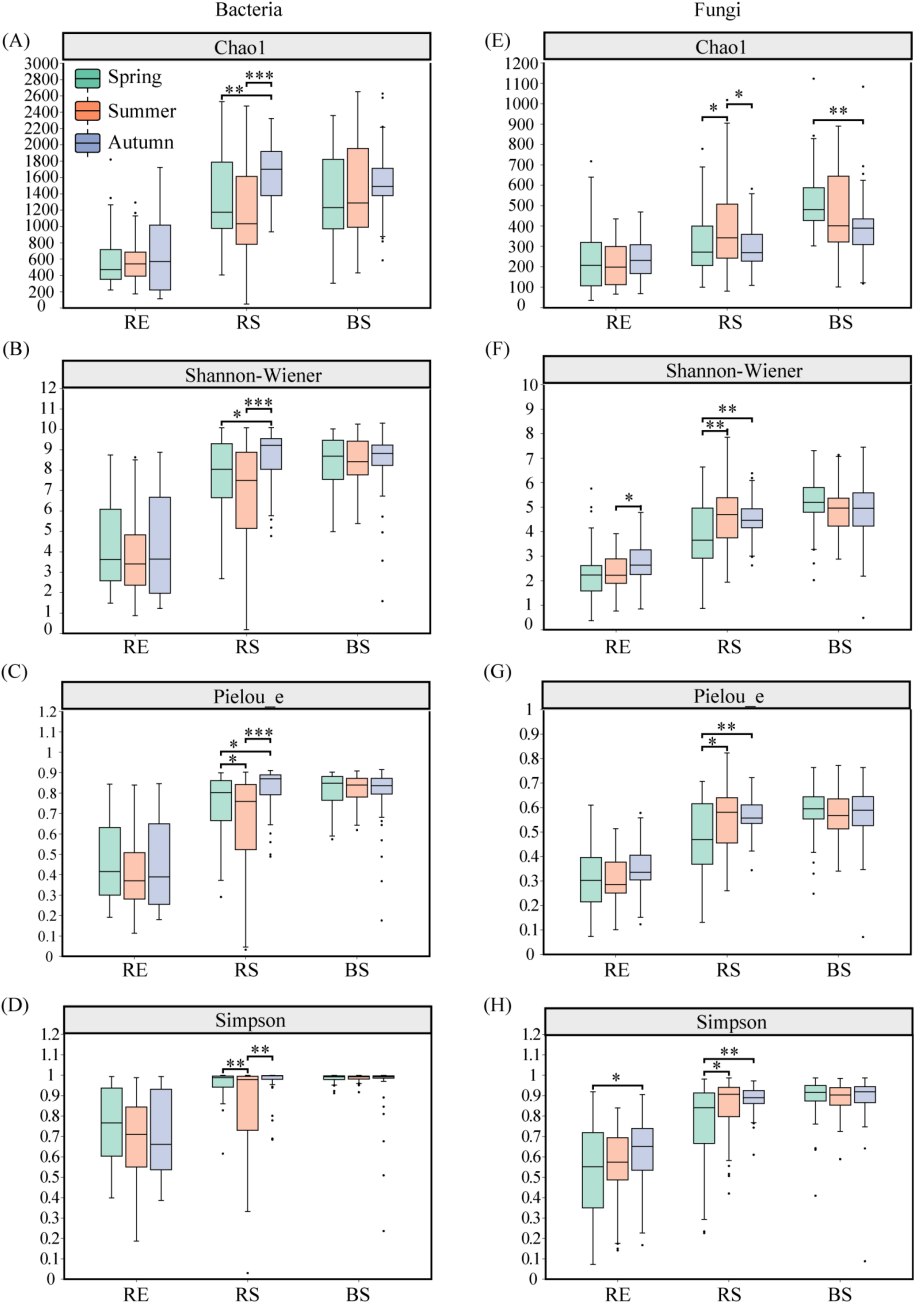
The α diversity of bacterial and fungal communities among season in desert plants. **(A–D)** the α diversity (Chao1, Shannon-Wiener, Pielou_e, and Simpson index) of bacterial communities among season in desert plants. **(E–H)** the α diversity (Chao1, Shannon-Wiener, Pielou_e, and Simpson index) of fungal communities among season in desert plants. RE, root endosphere; RS, rhizosphere soil; BS, bulk soil. Spring (May); Summer (July); and Autumn (September). Significance codes, *P < 0.05; **P < 0.01; ***P< 0.001.

The RS bacterial diversity (Chaol, Shannon-Wiener, and Pielou e) was notably higher in September when compared to May and July (*p* < 0.05). (**Figures 4A–C**). The bacterial Simpson index in RS was notably lower in July compared to May and September (*p* < 0.05) (**Figure 4D**). In July, the RS fungal Chaol index was significantly greater than in May and September (*p* < 0.05) (**Figure 4E**). Additionally, the RE fungal Shannon-Wiener index was notably higher in September relative to July (*p* < 0.05), and the Simpson index was significantly higher in September than in May (*p* < 0.05) (**Figures 4F, H**). TheRS fungal diversity (Shannon-Wiener, Pielou e, and Simpson) was significantly lower in May than in both July and September (*p* < 0.05). For BS fungi, the Chao1 index was notably higher in May compared to September (*p* < 0.05) (**Figures 4E–H**).

**Figure 5:**
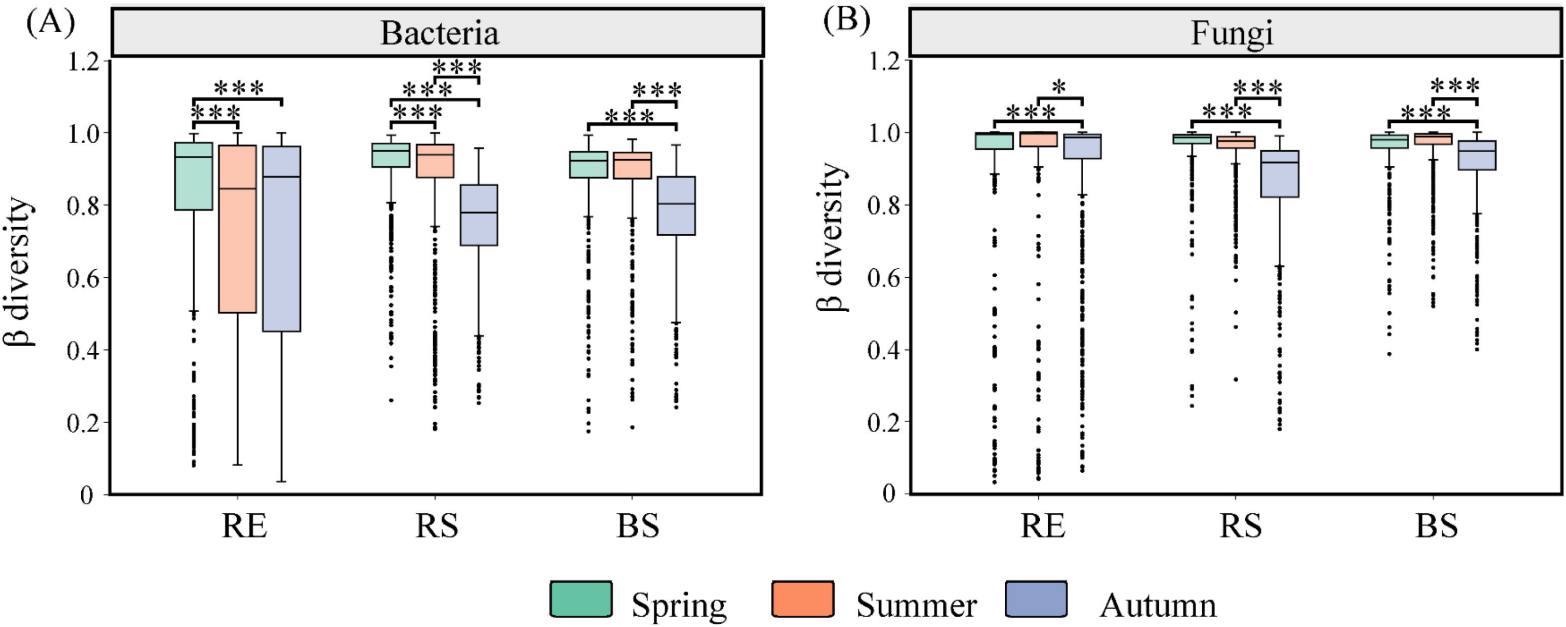
The β diversity of bacterial and fungal communities among season in desert plants. **(A)** the β diversity of bacterial communities among season in desert plants. **(B)** the β diversity of fungal communities among season in desert plants. RE, root endosphere; RS, rhizosphere soil; BS, bulk soil. Spring (May);Summer (July); and Autumn (September). Significance codes, *P < 0.05; ***P < 0.001.

The β diversity of RE, RS, and BS bacteria was significantly greater in May than in September (*p* < 0.05) (**Figure 5A**). In contrast, the β diversity of RE, RS, and BS fungi was significantly higher in July than in September (*p* < 0.05) (**Figure 5B**).

### 3.5 Seasonal variations influence the co-occurrence network of root microbial communities

**Figure 6:**
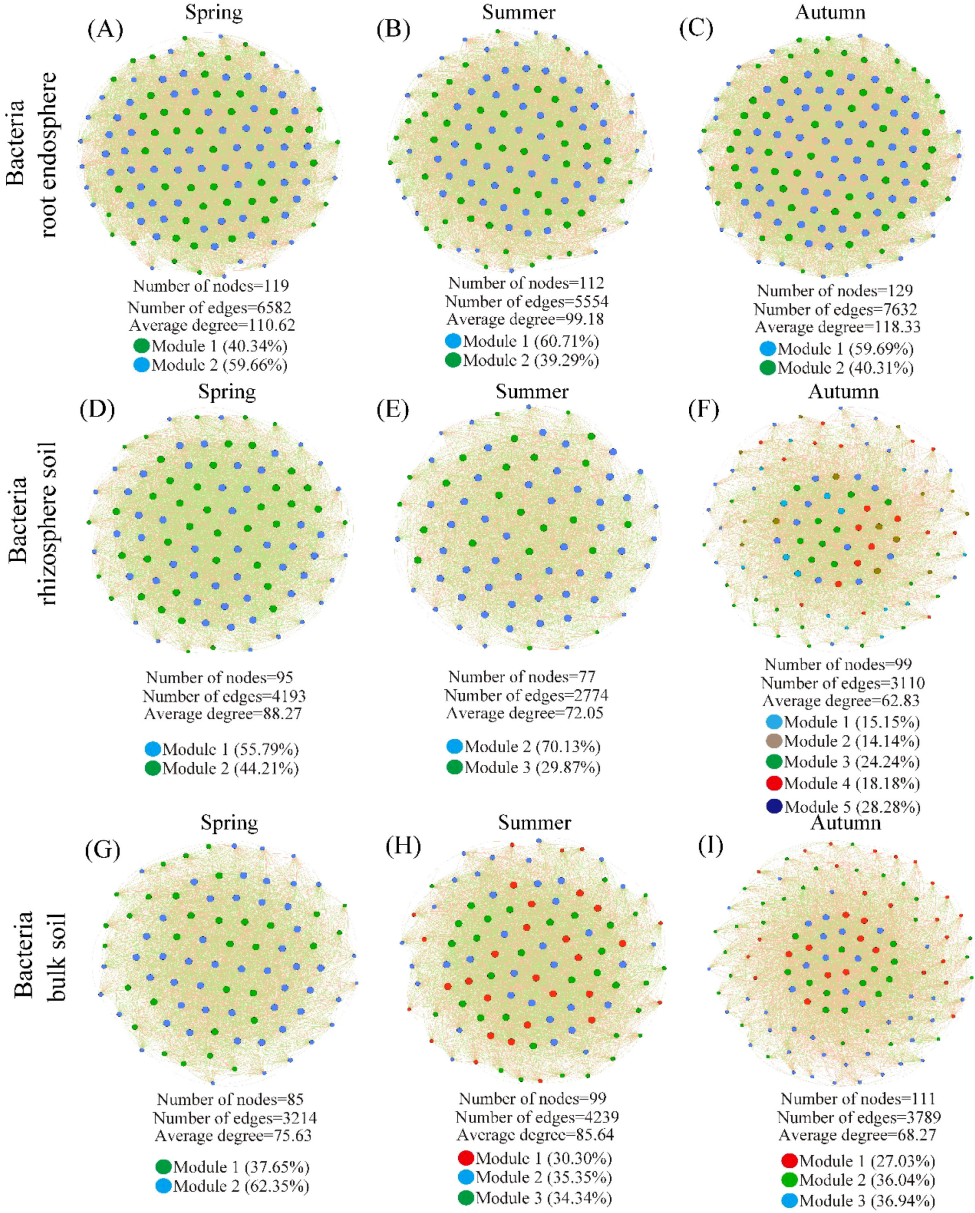
Co-occurrence network of root endosphere, rhizosphere soil, and bulk soil bacteria of desert plants. **(A–C)** theco-occurrence network of root endosphere bacteria of desert plants. **(D–F)** the co-occurrence network of rhizosphere soil bacteria of desert plants. **(G–I)** the co-occurrence network of bulk soil bacteria of desert plants. Spring (May); Summer (July); and Autumn (September).

In September, the RE, RS, and BS bacterial communities exhibited increased numbers of nodes, edges, and average degrees compared to May and July (**Figure 6**). In the fungal community, the number of nodes, edges, and average degree of RE and BS in September exceeded those recorded in May and July. The RS fungal nodes, edges, and average degree exhibited higher values in July compared to May and September (**Supplementary Figure S5**).

In May, the bacterial module connectivity mainly belongs to peripherals, and most of the fungal module connectivity belongs to connectors. In July, the bacterial modular connectivity includes peripherals and connectors, while the fungal modular connectivity includes peripherals. In September, both the bacteria and fungi module connectivity belong to peripherals and connectors (**Supplementary Figure S6**).

### 3.6 Effects of abiotic environmental factors on root microbial communities

**Figure 7:**
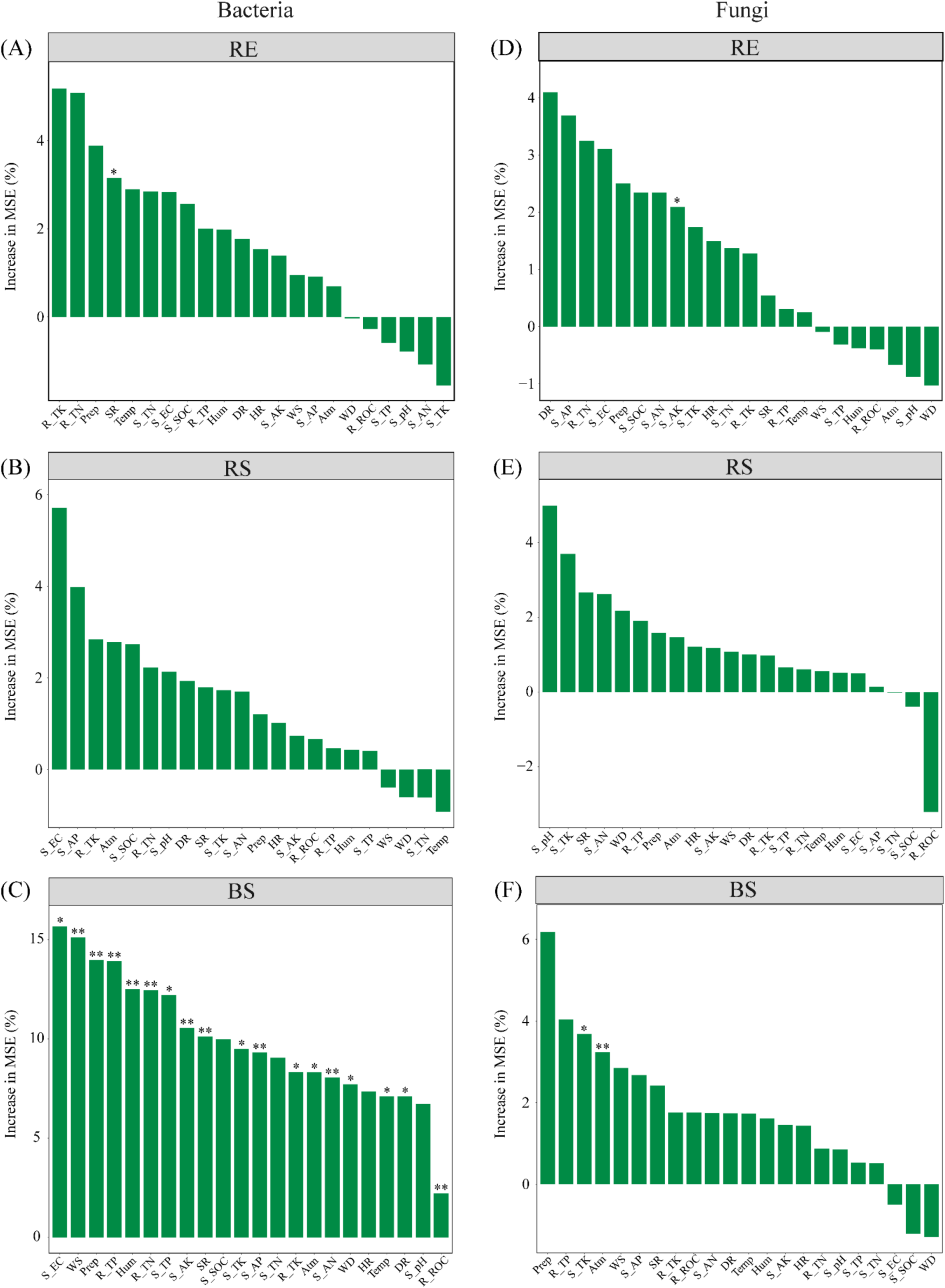
The random forest analyses between bacterial and fungal communities and environmental factors. **(A–C)** the random forest analyses between bacterial communities (RE, RS, and BS) and environmental factors. **(D–F)** the random forest analyses between fungal communities (RE, RS, and BS) and environmental factors. RE, root endosphere; RS, rhizosphere soil; BS, bulk soil; S_SOC, soil organic carbon (g•kg^-1^); R_ROC, root organic carbon (g•kg^-1^); TN, total nitrogen (g•kg^-1^); TP, total phosphorus (g•kg^-1^); TK, total potassium (g•kg^-1^); AN, available nitrogen (mg•kg^-1^); AP, available phosphorus (mg•kg^-1^); AK, available potassium (mg•kg^-1^); EC, electrical conductivity (mS•cm^-1^); Temp, temperature; Prep, precipitation; Atm, atmospheric pressure; WS, wind speed; WD, wind direction; DR, direct radiation; SR, scattered radiation; HR, horizontal radiation; Hum, air relative humidity. Significance codes, *P < 0.05; **P < 0.01.

Random forest analysis revealed that the variation of the RE bacterial community across different seasons was predominantly affected by SR (**Figure 7A**). The seasonal variation in the BS bacterial community was primarily influenced by WS, Prep, Hum, Atm, SR, WD, Temp, DR, soil properties (EC, TP, TK, AN, AP, and AK), and root nutrients (SOC, TN, TP, and TK), all of which had significant effects (**Figure 7C**). Seasonal changes in the RE fungal community were primarily affected by soil AK (**Figure 7D**). The seasonal variation in the BS fungal community was primarily affected by atmospheric pressure and soil TK (**Figure 7F**). The seasonal variation in the RS bacterial and fungal community has no significant influence factors (**Figures 7B, E**).

### 3.7 Pathway analysis of root microbial communities and abiotic factors

**Figure 8:**
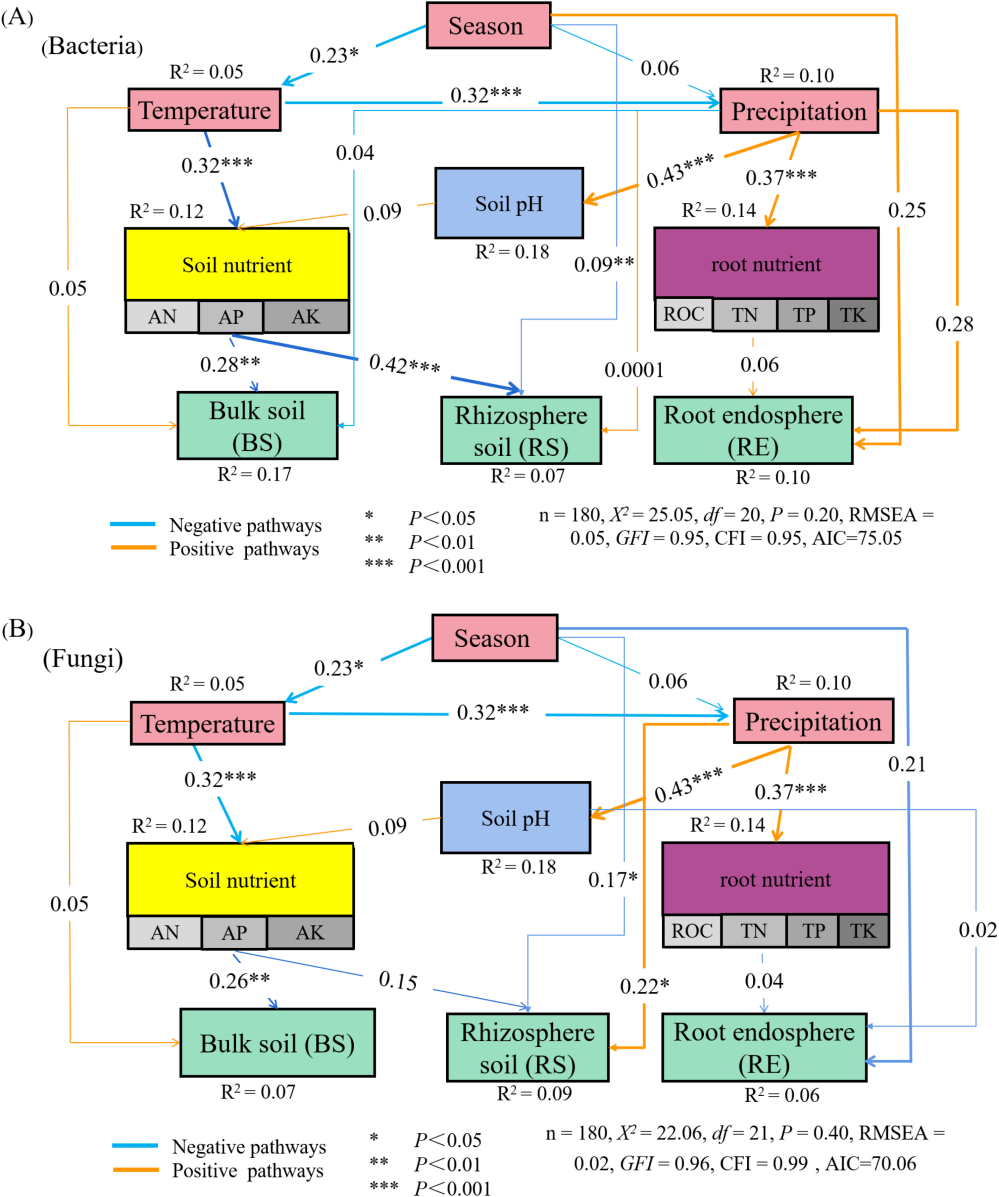
The structural equation model of effects of theenvironmental factors and soil or root nutrients on bacterial and fungal communities. **(A)** the structuralequation model of effects of theenvironmental factors and soil or root nutrients on bacterial communities. **(B)** the structural equation model of effects of theenvironmental factors and soil or root nutrients on fungal communities. ROC, root organic carbon (g•kg^-1^); TN, total nitrogen (g•kg^-1^); TP, total phosphorus (g•kg^-1^); TK, total potassium (g•kg^-1^); AN, available nitrogen (mg•kg^-1^); AP, available phosphorus (mg•kg^-1^); AK, available potassium (mg•kg^-1^).

Season exerted a notable negative direct influence on the RS bacterial community. It also had a significant negative direct effect on temperature, which in turn affected soil nutrient availability, consequently impacting microbial populations in both RS and BS compartments (**Figure 8A**). Similarly, season significantly and negatively affected the RS fungal community, while precipitation had a significant negative impact on the BS fungal community. Additionally, season directly and negatively influenced temperature, which significantly affected both precipitation and soil nutrients, thereby influencing fungal communities in RS and BS (**Figure 8B**). Regarding environmental drivers, the direct effect of precipitation on the RE bacterial community was stronger than that of temperature, soil nutrients, and root nutrients. Conversely, the total effect of precipitation on the RS fungal community surpassed those of temperature, soil nutrients, and root nutrients. For BS bacterial and fungal communities, temperature had a greater total effect than precipitation, soil nutrients, and root nutrients (**Table 6**).

**Table 6:** Path analysis of the root microbial community, physicochemical properties (of both root and soil), and climatic environmental factors.

Factor	Index	Root endosphere (RE)			Rhizosphere soil (RS)				Bulk soil (BS)		
		Direct effect	Indirect effect	Total effect	Direct effect	Indirect effect	Total effect	Direct effect		Indirect effect	Total effect
Bacteria	Season	0.25	0.0041	0.2541	-0.09	-0.031	-0.1211	0.00		-0.0323	-0.0323
	Temperature	0.00	0.00	0.00	0.00	0.00	0.00	0.05		0.00	0.05
	Precipitation	0.28	0.00	0.28	0.0001	0.00	0.0001	-0.04		0.00	-0.04
	Soil nutrient	0.00	0.00	0.00	-0.42	0.00	-0.42	-0.28		0.00	-0.28
	Root nutrient	0.06	0.00	0.06	0.00	0.00	0.00	0.00		0.00	0.00
	Soil pH	0.00	0.00	0.00	0.00	0.00	0.00	0.00		0.00	0.00
Fungi	Season	-0.21	-0.0003	-0.2103	-0.17	-0.0243	-0.1943	0.00		-0.0308	-0.0308
	Temperature	0.00	0.00	0.00	0.00	0.00	0.00	0.05		0.00	0.05
	Precipitation	0.00	0.00	0.00	0.22	0.00	0.22	0.00		0.00	0.00
	Soil nutrient	0.00	0.00	0.00	-0.15	0.00	-0.15	-0.26		0.00	-0.26
	Root nutrient	-0.04	0.00	-0.04	0.00	0.00	0.00	0.00		0.00	0.00
	Soil pH	-0.02	0.00	-0.02	0.00	0.00	0.00	0.00		0.00	0.00

## 4 Discussion

### 4.1 Effects of seasonal variation on the composition and stability of root bacterial and fungal community

This study found that *Proteobacteria* and *Cyanobacteria* were the core bacterial phyla in the root communities of typical desert plants across seasonal changes, while *Ascomycota* was the dominant fungal phylum. Notably, the RE bacterial community contained a higher abundance of *Cyanobacteria*, whereas its fungal community exhibited a lower proportion of *Ascomycota*. Due to the arid climate, infrequent precipitation, low soil moisture content, and poor nutrients in desert areas (Zhang et al., 2022b; Islam et al., 2024a), plant roots provide suitable living places and nutrients for *Cyanobacteria*, in turn, they provide nitrogen and other nutritional support for plants (Zhang et al., 2024a; Zhao et al., 2024). This mutually beneficial symbiotic relationship is conducive to the colonization and reproduction of the *Cyanobacteria*, however, desert plants are also more inclined to establish close ties with *Cyanobacteria*, which provides favorable conditions for its survival in roots (Zhang et al., 2024a; Zhao et al., 2024). Studies indicate that fungal communities in roots are vital for grass resource acquisition, particularly in arid grasslands where *Ascomycota* predominates (Green et al., 2008; Porras-Alfaro et al., 2008). *Ascomycota* is vital for collaborating with other microbes to decompose soil litter, increase nutrient availability, support plant growth, and adapt to harsh environments (Glassman et al., 2017; Islam et al., 2024b).

In this study, the relative abundance of *Ascomycota* in RE was lower than that in RS and BS. It indicates that the selection of microorganisms within the RE has a strong host selection effect that root epidermis providing a more stable and buffered habitat (Maestre et al., 2015; Bahram et al., 2018; Xiong et al., 2021). Numerous studies have found that certain microorganisms in the rhizosphere attach to and are filtered by the roots, ultimately influencing the aggregation of the microbial community in the roots (Edwards et al., 2018). Additionally, roots not only attract microorganisms nearby but also possess genetically determined factors that contribute to the stability of specific microbial species (Bulgarelli et al., 2013). Our study also found that the community assembly of bacteria and fungi in both RE and RS associated with desert plants across different seasons primarily followed a stochastic process, mainly driven by dispersal limitation. In desert regions, frequent high temperatures during July (summer) and September (autumn) lead to elevated soil surface temperatures (Zhang et al., 2022b; Islam et al., 2024c). To protect themselves from such environmental stresses, desert plant roots release exudates that recruit specialized microorganisms, which bind tightly to the roots forming protective complexes (Haichar et al., 2008; Reinhold-Hurek et al., 2015). While our results confirm that environmental factors influence the recruitment of soil microbes to the rhizosphere (Fitzpatrick et al., 2018; Wu et al., 2021), it is crucial to recognize the importance of host-specific inheritance in maintaining microbial community stability in desert plants.

Co-occurrence network analysis revealed that in May (spring), root bacterial communities lacked highly connected nodes both within and between modules. In contrast, such highly connected nodes were present in July (summer) and September (autumn). This observed pattern could potentially be attributed to the seasonal variations in temperature and precipitation, as well as the pronounced micro-environmental gradient from the RE to BS, which is mainly influenced by soil pH, water availability, and nutrients (Hinsinger et al., 2009; Ali et al., 2024; Zhang et al., 2024b). Except as microbial co-occurrence networks, which can characterize the complexity and stability of microbial communities, the microbial diversity index can indicate the degree of complexity among them (Coyte et al., 2015; de Vries et al., 2018; Maurice et al., 2024b). Seasons had a significant influence on the α diversity of root bacteria and fungi. However, the β diversity of root bacteria and fungi in desert plants decreased significantly in autumn. Phreatophytes in desert ecosystems exhibit long-term adaptability to external conditions, leading to varied outcomes (Hernandez et al., 2021; Islam et al., 2024a). Our findings showed that the RS bacterial diversity (Chao1, Shannon-Wiener, and Pielou_e) was significantly higher in September (autumn) compared to May (spring) and July (summer). On the one hand, the plants grow vigorously in summer, and rhizosphere microorganisms compete with each other to obtain limited carbon sources, nitrogen sources, and other nutrients. The preferential elimination by rhizosphere microorganisms decreases their β diversity (Lai et al, 2025). On the other hand, the high temperature and arid environment in autumn may destroy the established symbiotic relationship between rhizosphere microorganisms, which may not only affect the types of microorganisms involved in symbiosis but also hurt the structure and diversity of the entire rhizosphere microbial community (Berendsen et al., 2012; Durán et al., 2018; Xie and Yin, 2022).

### 4.2 Influence of environmental factors on root bacterial and fungal community composition and diversity

Our study revealed that soil total potassium content was significantly higher in July (summer) and September (autumn) compared to May (spring), likely due to increased plant growth and accumulation of plant residues during these seasons, which are returned than in spring. These plant residues contain a certain amount of potassium, which reenters the soil after decomposition (Dawson and Goldsmith, 2018; Gao et al., 2023). The root organic carbon content in July (summer) is significantly higher than in May (spring). This is because there is less precipitation in desert areas in July (summer) (Gao et al., 2023; Zhang et al., 2024b). To absorb and store water more effectively, plants tend to develop more developed roots, which also promotes the increase of root organic carbon content (Körner, 2015; Ledo et al., 2020). In this study, the RE bacterial communities were mainly influenced by scattered radiation in different seasons. For the BS bacterial community, its seasonal variation was mainly caused by climate and was significantly affected by soil physicochemical properties and root nutrients. Regarding the RE and BS fungal communities, their seasonal variations were mainly influenced by soil nutrients. Therefore, it can be concluded that the root microbial composition and diversity (RE, RS, and BS) in deep-rooted desert plants are influenced by factors beyond just soil and root nutrients. Instead, climatic factors from the external environment are likely to have the most direct impact on these microbial communities (Trivedi et al., 2022).

Environmental changes influence the spatial heterogeneity of microbial communities, with environmental factors playing a significant role in shaping the assembly processes of rhizosphere and root endosphere microbial communities (Chase and Myers, 2011; Zhong et al., 2022). Seasonal variations in precipitation have a direct positive effect on the composition of the RS fungal community. Conversely, these precipitation changes indirectly inhibit the composition of the BS fungal community by reducing the availability of soil nutrients. Season, temperature, and precipitation indirectly impact RE and BS fungal communities by affecting soil and root nutrient availability. Many studies have demonstrated that climate factors contribute substantially, accounting for 65% of soil fungal community fluctuations in the biogeography of gramineous root-associated fungi in the North American Plains (Hawkins et al., 2003; Jennifer et al., 2022). Studies have shown that the relationship between the fungal communities in plant roots and soil is not close but rather dispersed across plant and soil compartments, which helps maintain microenvironmental stability (Chase, 2007; Glynou et al., 2018). The changes in precipitation and temperature caused by seasonal variations can indirectly and significantly inhibit the community composition of RS and BS bacteria by significantly altering soil pH and available nutrients. Previous studies have confirmed that precipitation and soil pH have a significant influence on the diversity and structure of soil microbial communities at both regional and global levels (Maestre et al., 2015; Bahram et al., 2018; Delgado-Baquerizo et al., 2018). Climatic and soil factors affect the composition of soil microbiota and the attraction of microorganisms to the rhizosphere via plant roots (Philippot et al., 2013; Edwards et al., 2015; Makhalanyane et al., 2015).

## 5 Conclusion

Seasonal variation exerted a significant influence on the α diversity of both bacterial and fungal communities in desert plant-associated environments. Specifically, within the root endosphere (RE), rhizosphere soil (RS), and bulk soil (BS), the β diversity of bacterial and fungal communities was markedly higher during May (spring) and July (summer) compared to September (autumn), indicating a pronounced seasonal shift in microbial composition. This pattern suggests that spring and summer conditions favor greater microbial differentiation, possibly due to more dynamic environmental changes and plant activity during these periods. The diversity and structure of root-associated microbial communities were shaped by a complex interplay of factors, including soil physicochemical properties, plant-derived nutrient inputs, and external climatic variables such as temperature and precipitation. Notably, the fungal community within the RE was found to be directly influenced by seasonal shifts, whereas the RS fungal community composition was significantly impacted by changes in precipitation patterns driven by seasonal variation. In contrast, temperature showed only a limited effect on fungal community composition across the three compartments (RE, RS, and BS), highlighting the stronger role of moisture availability and nutrient cycling. These findings contribute important insights into the seasonal dynamics of microbial assemblages in desert ecosystems and help address existing knowledge gaps concerning the ecological roles and habitat specificity of microbes associated with deep-rooted desert plants.

## Data Availability

The datasets presented in this study can be found in online repositories. The names of the repository/repositories and accession number(s) can be found in the article/**Supplementary Material**.
