# Supplementary material for: Impact of seasonal changes on root-associated microbial communities among phreatophytes of three basins in desert ecosystem
Source: Front Plant Sci. 2025 Jul 4;16:1554879. doi: 10.3389/fpls.2025.1554879 (PMC12272231; doi:10.3389/fpls.2025.1554879)

**Impact of Seasonal Changes on Root-Associated Microbial Communities among Phreatophytes of Three Basins in Desert Ecosystem**

**Yulin Zhang^a,b,c,e#^, Yi Du^a,b,c,d#^, Zhaobin Mu^a,b,c,g*^, Waqar Islam^a,b,c^*, Fanjiang Zeng^a,b,c,d,e^*, Norela C. T. Gonzalez^f^, Zhihao Zhang^a,b,c^**

^a^ Xinjiang Key Laboratory of Desert Plant Roots Ecology and Vegetation Restoration, Xinjiang Institute of Ecology and Geography, Chinese Academy of Sciences, Urumqi 830011, China

^b^ State Key Laboratory of Ecological Safety and Sustainable Development in Arid Lands, Xinjiang Institute of Ecology and Geography, Chinese Academy of Sciences, Urumqi 830011, China

^c^ Cele National Station of Observation and Research for Desert-Grassland Ecosystems, Cele 848300, China

^d^ University of Chinese Academy of Sciences, Beijing 100049, China

^e^ College of Ecology and Environmental, Xinjiang University, Urumqi 830046, China

^f^ College of Forestry, Central South University of Forestry and Technology, Changsha 410004, Hunan, China

^g^ State Key Laboratory of Advanced Environmental Technology and Guangdong Key Laboratory of Environmental Protection and Resources Utilization, Guangzhou Institute of Geochemistry, Chinese Academy of Sciences, Guangzhou 510640, China

#Y.L. Zhang and Y. Du contributed equally to this work.

* Corresponding author.

E-mail addresses: [muzhaobin@ms.xjb.ac.cn](mailto:muzhaobin@ms.xjb.ac.cn) (**Zhaobin Mu**)

waqarislam@ms.xjb.ac.cn (**Waqar Islam**)

fjzeng@ms.xjb.ac.cn (**Fanjiang Zeng**)

Table S1 Variance analysis of alpha diversity in typical desert plants by different seasons and root partitioning

|  | Phylum | Season | Root partitioning | Season×Root partitioning |
| --- | --- | --- | --- | --- |
| Bacteria | *Proteobacteria*  *Cyanobacteria* | 5.18**  1.05 | 15.40***  216.18*** | 5.27***  2.02 |
|  | *Actinobacteriota* | 12.00*** | 18.32*** | 0.74 |
|  | *Firmicutes* | 0.80 | 25.81*** | 1.38 |
|  | *Bacteroidota*  *Chloroflexi*  *Gemmatimonadota*  *Deinococcota*  *Acidobacteriota*  *Verrucomicrobiota* | 7.53***  3.15*  1.66  4.35*  1.53  2.64 | 43.42***  76.08***  61.34***  19.31***  34.78***  29.71*** | 1.92  4.26**  2.81*  2.99*  3.63**  3.02* |
| Fungi | *Ascomycota*  *Fungi_phy_Incertae_sedis* | 2.13  1.67 | 85.12***  6.50** | 1.03  4.90*** |
|  | *Basidiomycota* | 6.66*** | 21.99*** | 2.55* |
|  | *Chytridiomycota* | 0.60 | 6.29** | 0.34 |
|  | *Mucoromycota*  *Mortierellomycota*  *Blastocladiomycota*  *Aphelidiomycota*  *Rozellomycota*  *GS01_phy_Incertae_sedis* | 2.44  3.95*  1.96  2.10  1.28  0.55 | 2.21  9.75***  2.68  4.87**  5.09**  2.49 | 21.47  2.74*  0.90  1.17  2.08  0.63 |

**Note:** * *P* < 0.05; ** *P* < 0.01; *** *P* < 0.001.

**Figure legends**

**Fig. S1** The three sampling sites at Cele, Turpan, and Mosuowan are located in Tarim Basin, Turpan Basin, and Junggar Basin, respectively.

**Fig. S2** The dilution curve of bacterial and fungal communities in desert plants.

**Fig. S3** Mantel correlogram based on phylogenetic distance and environmental euclidean distances of root-associated (RE, RS, and BS) bacterial and fungal ASV (Amplicon Sequence Variants) **RE**, root endosphere; **RS**, rhizosphere soil; **BS**, bulk soil.

**Fig. S4** The absolute value of normalized effect size (SES) of bacterial and fungal communities of desert plants across the seasons. **RE**, root endosphere; **RS**, rhizosphere soil; **BS**, bulk soil.

Note: The values of observed C-score（C-scoreob, green）> simulated C-score（C-scoresim, orange）show nonrandom co-occurrence patterns. Standardized effect size（SES, red squares）< 2 and >2 represent aggregation and segregation respectively.

**Fig. S5** Co-occurrence network of root endosphere, rhizosphere soil, and bulk soil fungi of desert plants.

**Fig. S6** Network connectivity of bacterial and fungal communities of desert plants.

**Fig. S1**

**
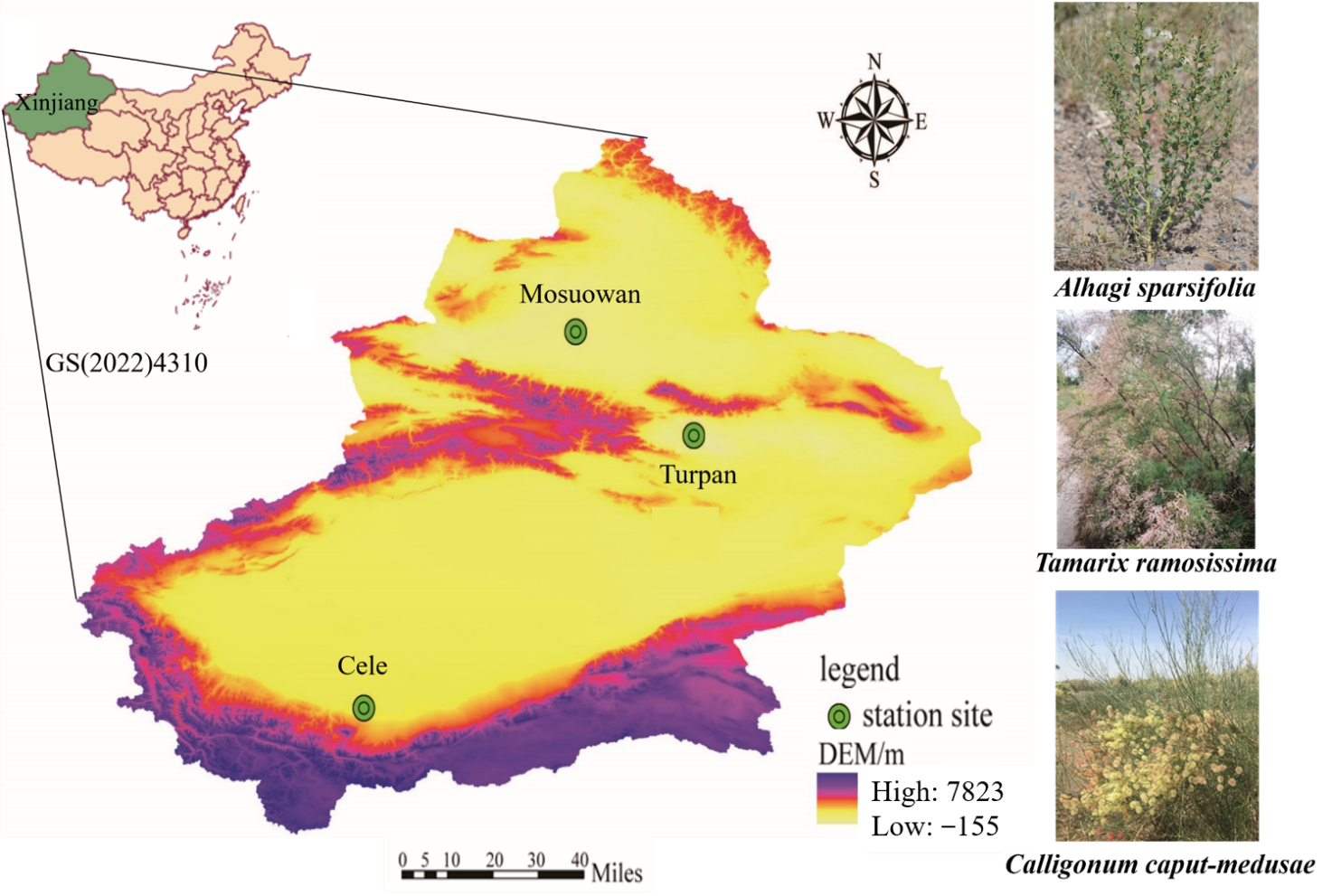
**

**Fig. S2**


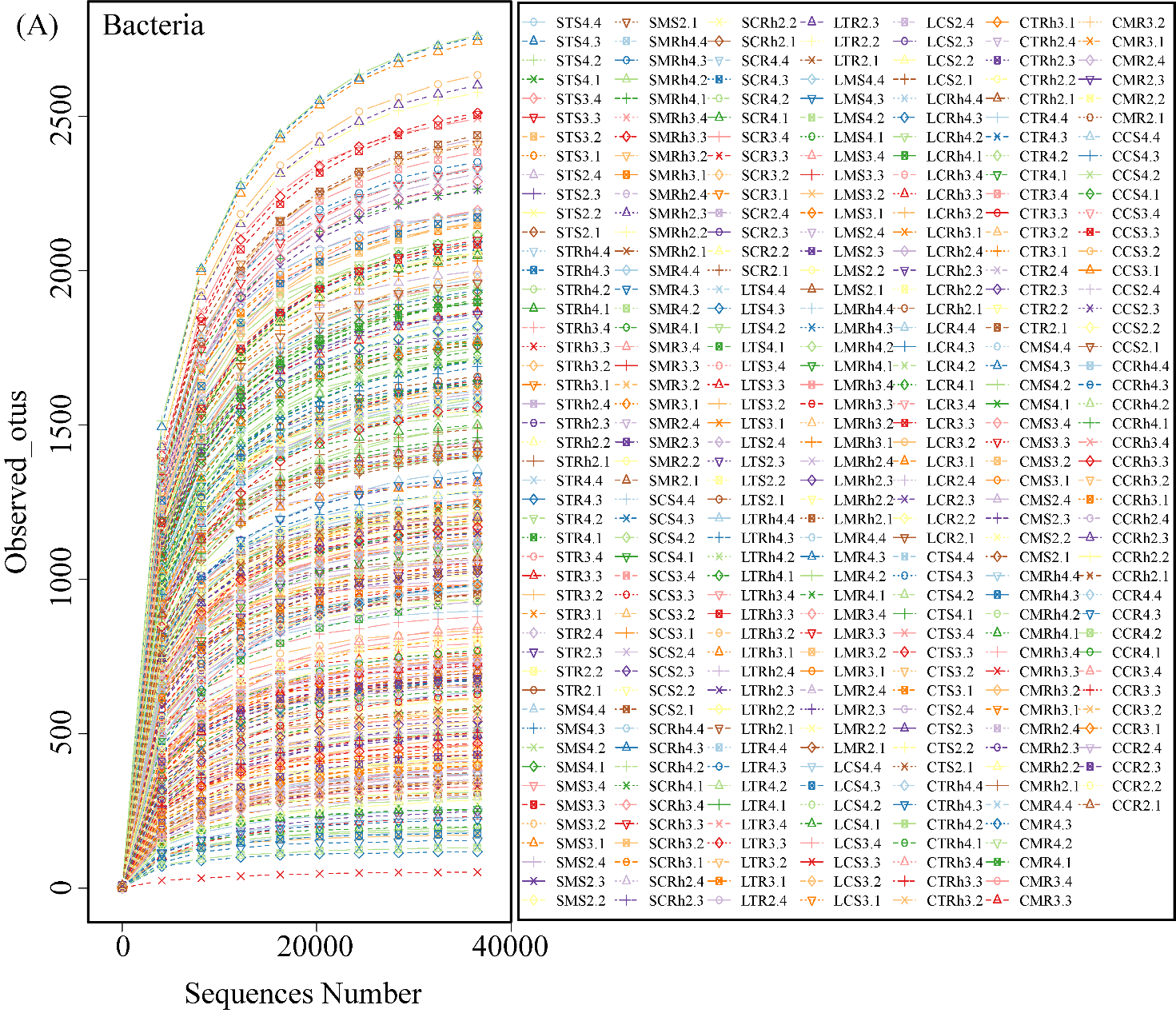


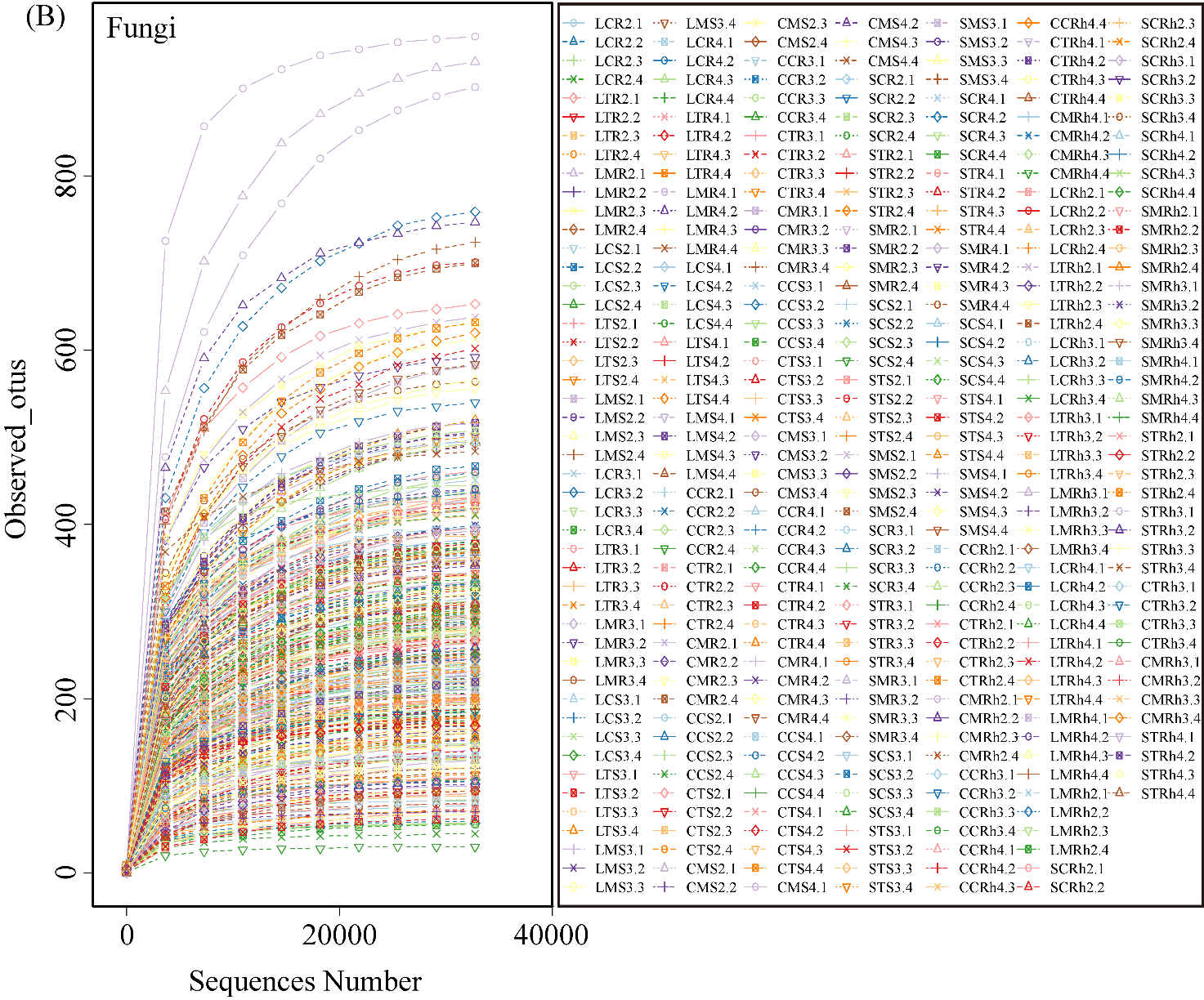


**Fig. S3**


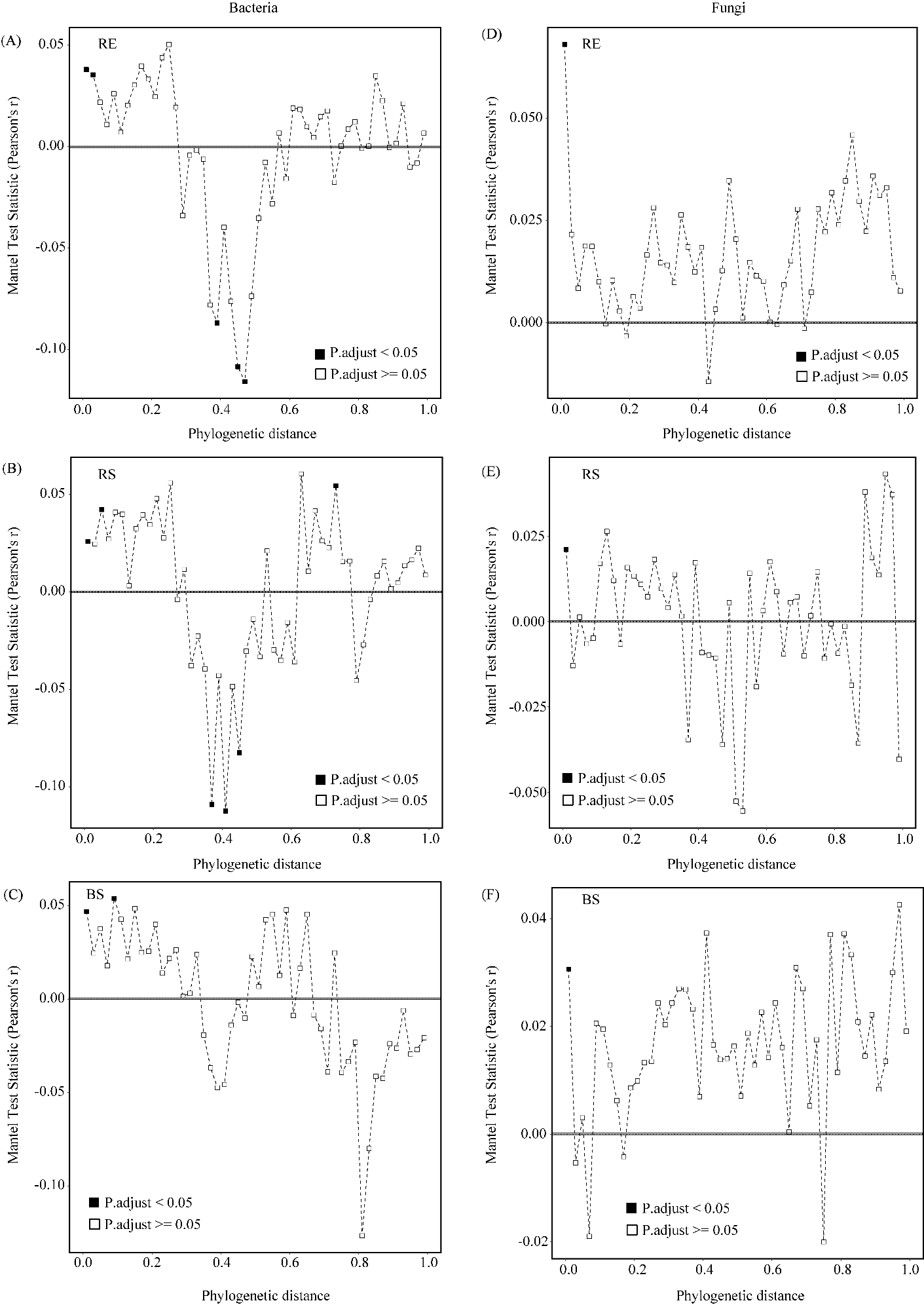


**Fig. S4**


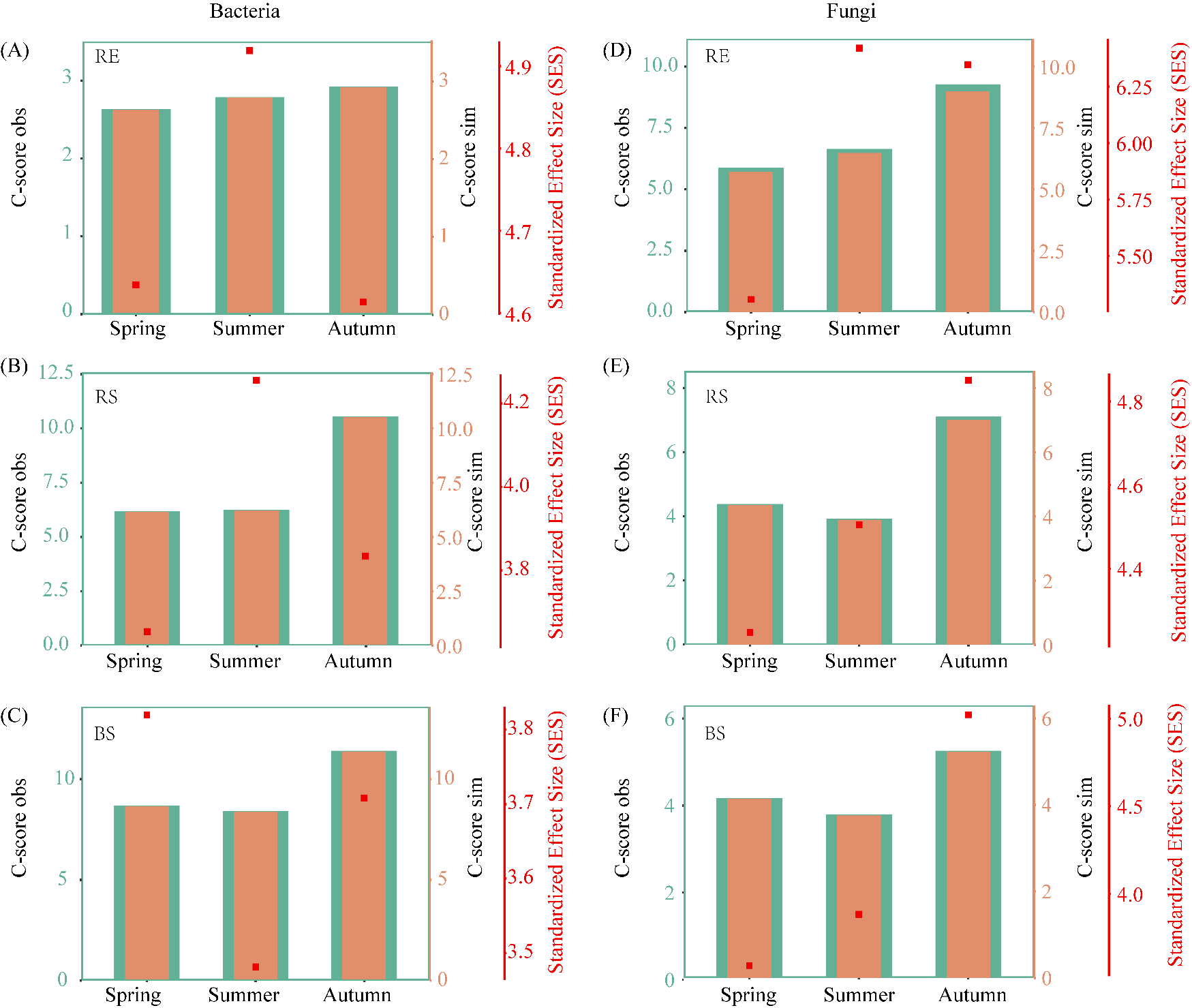


**Fig. S5**


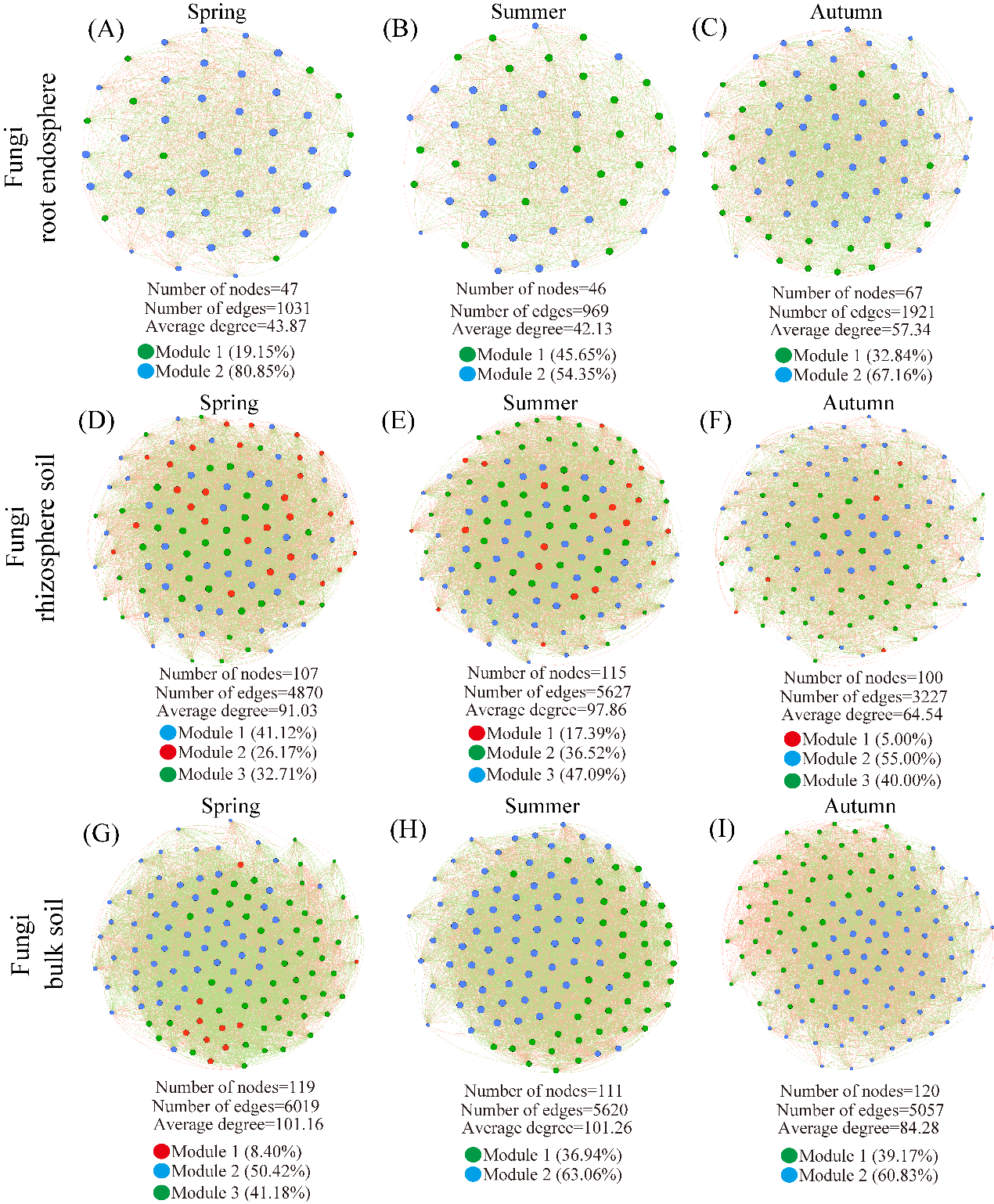


**Fig. S6**


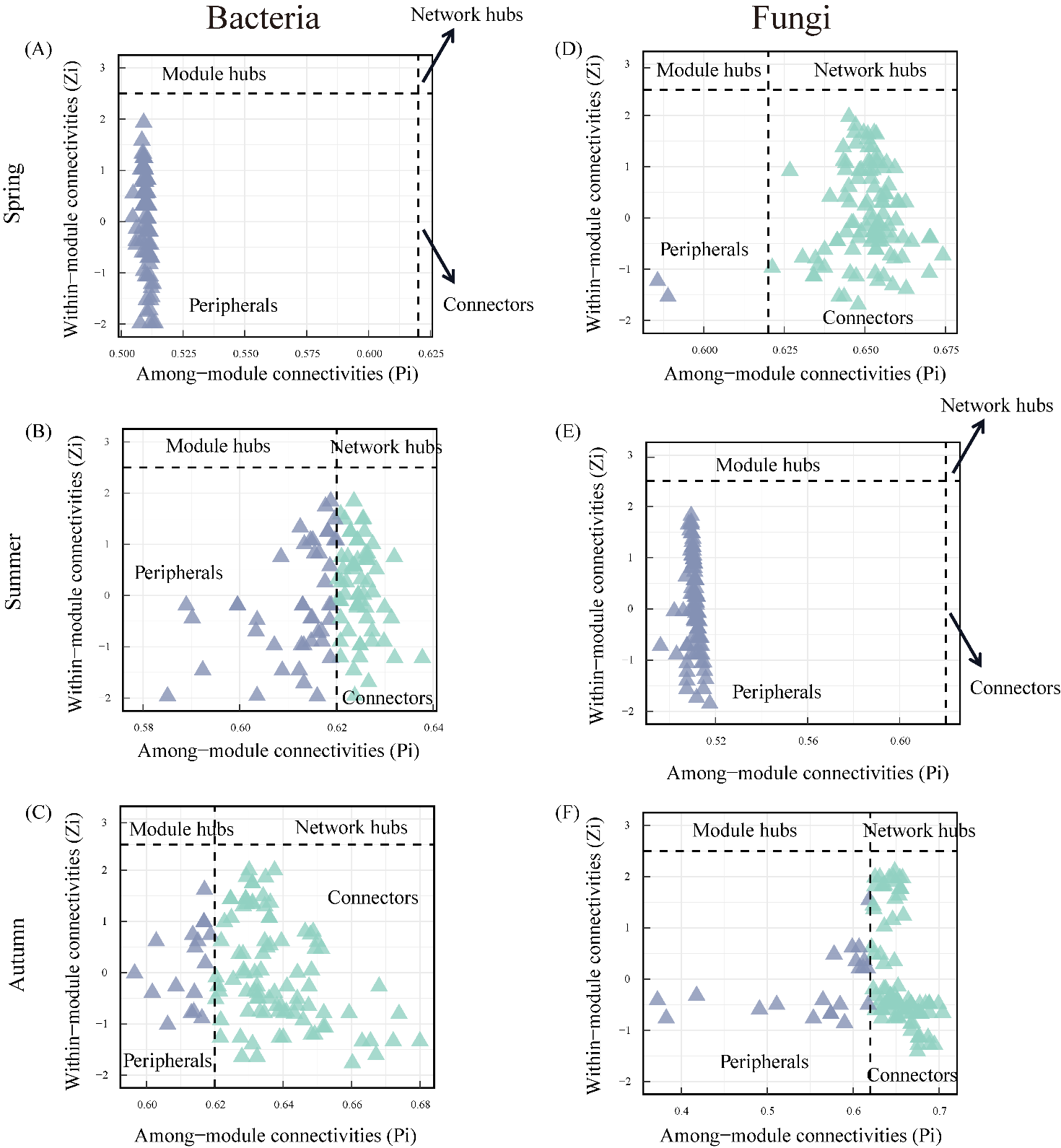

Supplement: Supplementary file 1 [file DataSheet1.docx]
